# Scope of Message Planning: Evidence From Production of Sentences With Heavy Sentence‐Final NPs

**DOI:** 10.1111/cogs.70110

**Published:** 2025-10-14

**Authors:** Agnieszka E. Konopka

**Affiliations:** ^1^ School of Psychology University of Aberdeen

**Keywords:** Message planning, Sentence planning, Incrementality, Modification, Eye movements

## Abstract

Speaking begins with the generation of a preverbal message. While a common assumption is that the scope of message‐level planning (i.e., the size of message‐level increments) can be more extensive than the scope of sentence‐level planning, it is unclear how much information is typically encoded at the message level in advance of sentence‐level planning during spontaneous production. This study assessed the scope and granularity of early message‐level planning in English by tracking production of sentences with light versus heavy sentence‐final NPs. Speakers produced SVO sentences to describe pictures showing an agent acting on a patient. Half of the pictures showed one‐patient events, eliciting sentences with unmodified patient names (e.g., “*The tailor is cutting the dress*”), and half showed two‐patient events with a target patient and a non‐target patient. The presence of a non‐target patient required production of a prenominal or postnominal modifier to uniquely identify the target patient (e.g., “*The tailor is cutting the long dress*” / “*the dress with sleeves*”). Analyses of speech onsets and eye movements before speech onset showed strong effects of the complexity of the sentence‐final character, suggesting that early message‐level planning does not proceed strictly word by word (or “from left to right”) but instead includes basic information about the identity of both the sentence‐initial and sentence‐final characters. This is consistent with theories that assume extensive message‐level planning before the start of sentence‐level encoding and provides new evidence about the level of conceptual detail incorporated into early message plans.

Producing an utterance requires engaging in two broad classes of processes (Levelt, 1989): deciding *what* to say (message‐level or conceptual planning) and *how* to say it (sentence‐level or linguistic planning). Messages are defined as prelinguistic, nonlinear representations that are generated for the purpose of communication and that may be, to some extent, influenced by properties of the target language (Levelt, 1989, 1999). The ways in which the *content* of messages can differ cross‐linguistically are still debated. Regardless of the target language, however, a common feature of the message planning *process* during spontaneous production in any language is that these representations are generated dynamically and that planning scope (i.e., the size of planning increments or chunks—the amount of information that is planned in parallel at each level of encoding at any given moment in time) can vary in response to a number of production pressures. A puzzle tackled by numerous psycholinguistic studies concerns the scope of advance planning at the level of messages and sentences across production contexts, as well as the factors that explain variability in planning scope.

The overall consensus is that production is incremental: speakers can prepare both their messages and sentences in chunks (or increments) that are smaller than the utterance they will eventually produce, and they can continue preparing new chunks of the same message and sentence while production of earlier chunks is underway (Levelt, 1989). Importantly, planning scope (i.e., increment size) at the message level and at the sentence level need not coincide. It is commonly assumed that the scope of planning at higher levels can be more extensive than the scope of planning at lower levels: sentence‐level chunks can be phrasal or subphrasal (e.g., Konopka, 2012; Martin, Crowther, Knight, Tamborello II, & Yang, 2010; Meyer, 1996; Smith & Wheeldon, 1999), but message‐level chunks can vary in size from chunks that correspond to individual lexical items (e.g., Brown‐Schmidt & Konopka, 2008) to chunks that consist of multiple concepts that will eventually be produced in different phrases (e.g., Griffin & Bock, 2000). The upper bound of these larger message‐level chunks has been notoriously hard to define, and, in fact, existing accounts do not specify how much conceptual detail must be encoded at the message level in cases where message‐level planning does run ahead of sentence‐level planning. A closely related puzzle concerns the order in which message‐level and sentence‐level information is encoded, both across and within languages. While planning at lower levels can proceed in an order corresponding to linear word order (i.e., word by word or “from left to right”^1^), it is unclear to what extent planning of multiple concepts at the message level can occur in parallel and whether it is shaped by a similar “left‐to‐right” bias (see Konopka & Brown‐Schmidt, 2014, and Bock & Ferreira, 2014, for reviews). The current paper addresses this question, for the first time, by comparing planning of messages and sentences with light and heavy sentence‐final NPs. The target language is English, i. e., a language with relatively rigid word order, limited case marking, and a strong preference for SVO syntax.

## 1 Incrementality in message‐level planning

The hypothesized lower and upper bounds of message‐level increments are described by two competing accounts of incremental planning: Linear and Hierarchical Incrementality. For example, generating an active SVO sentence in English to describe a pictured event in which a thief is stealing a painting (“The thief is stealing the painting”) may proceed in at least two different ways (Griffin & Bock, 2000; Hwang & Kaiser, 2014, 2015; Konopka & Meyer, 2014; Konopka, Meyer, & Forest, 2018; Kuchinsky & Bock, 2010; Norcliffe et al., 2015; Sauppe, 2017; Nordlinger, Rodriguez, & Kidd, 2022). Under the strong version of Linear Incrementality, planning scope may be highly restricted, with the preverbal message being prepared in small chunks that are eventually concatenated to form a longer message: speakers may first encode a message‐level increment consisting of as little information as a single character (*thief*)—likely the most perceptually or conceptually salient character at the moment of speaking (e.g., Gleitman, January, Nappa, & Trueswell, 2007; Myachykov, Thompson, Scheepers, & Garrod, 2011)—and may then plan and add subsequent conceptual chunks to the developing message sequentially on the fly (see Brown‐Schmidt & Konopka, 2008, 2015). In this example, message‐level planning does not “outrun” sentence‐level planning: speakers can encode the concept *thief* and retrieve the corresponding noun “thief,” then encode the concept *steal* and retrieve the corresponding verb “steal,” and finally encode the concept *painting* and retrieve the corresponding noun “painting” (with some overlap between sentence‐level encoding of each word and message‐level planning of the next concept). Articulation of each chunk can start as soon as that chunk is encoded linguistically (“*The thief*…,” “…*is stealing*…,” “…*the painting*”). In such a radically incremental system, the order in which message‐level information is encoded corresponds to or is compatible with the linear “left‐to‐right” order in which sentence‐level information is eventually produced. In contrast, the strong version of Hierarchical Incrementality posits that message‐level scope is much wider and thus that message‐level planning does substantially “outrun” sentence‐level planning: encoding of a message begins with the generation of a nonlinear representation—that is, a broad relational who‐did‐what‐to‐whom framework for the event (a theft involving a man and a painting)—that can incorporate as much information as is eventually expressed with an entire sentence (see Konopka & Brown‐Schmidt, 2014, and Bock & Ferreira, 2014, for reviews). This information is viewed as being part of a single larger message‐level increment and is then passed on to sentence‐level planning processes in one step (with articulation of each sentence‐level chunk starting as soon as that chunk is encoded). The upper bound of message‐level increments is, therefore, much higher under Hierarchical Incrementality than under Linear Incrementality.^2^

Any account assuming a high upper bound to message‐level increments leaves open two related questions. The first question concerns the size of these increments: if planning involves generation of a relational framework that, in the current example requires planning of several conceptual elements (i.e., the “who,” “did what,” and “to whom” elements of the message), how much detail is encoded about *each* element during early message planning? The second question concerns the timing of the mapping between message‐level and sentence‐level increments. In principle, multiple conceptual elements may be encoded in parallel at the message level, but because words must ultimately be produced in a specific order, the mapping of information from a nonlinear message representation onto a linear linguistic representation requires that word order be specified at some stage during planning. This mapping—a critical process at the interface of “thinking” and “speaking”—remains understudied (Bock & Ferreira, 2014).

A plausible solution to the mapping problem is that the process of message planning itself may involve some degree of linearity (i.e., a “left‐to‐right” bias). There is now ample evidence that, when producing sentences describing multi‐character events, speakers are able to plan more than one concept at the same time at the message level (Konopka & Meyer, 2014; Konopka et al., 2018; Konopka, 2019; Kuchinsky & Bock, 2010; Norcliffe, Konopka, Brown, & Levinson, 2015; Nordlinger et al., 2022; Sauppe, 2017), but that the order in which these concepts are planned can be influenced by two encoding biases: a bias to encode relational information early and a bias to encode agents early.

The proposal that message planning starts with the generation of a who‐did‐what‐to‐whom framework implies that relational information must be encoded early with priority (Griffin & Bock, 2000; Bock & Ferreira, 2014). In the current example, this includes information about the action performed by the agent on the patient (i.e., the “did what” element of the relational framework), which is then expressed at the sentence level with a specific verb (Konopka, 2019).^3^ In the lab, tracking speakers’ eye movements during spontaneous sentence production tasks has shown clear sensitivity to the ease of encoding relational information in an event from the earliest stages of planning. Speakers typically fixate the areas of the visual displays that include the information that is most relevant for in‐the‐moment encoding. While the encoding of character‐specific information can take place by directing attention to individual characters, relational information is typically “distributed” across characters and must be encoded by processing action‐relevant details of both characters. Thus, the eye movement signature of early relational encoding is, arguably, a pattern of fixations alternating between agents and patients shortly after picture onset (Griffin & Bock, 2000), with a preference for the character that has more features relevant for encoding the event action (Konopka, 2019). Early eye movements are also influenced by the ease of expressing the event action, as indexed by a measure of verb agreement in speakers’ descriptions (event codability: Kuchinsky & Bock, 2010). For languages where verbs are not produced in sentence‐initial position (like English), such evidence suggests that encoding priorities are not defined exclusively by linear word order and is, therefore, consistent with the view that message‐level representations are, at least to some extent, nonlinear in nature.

Interestingly, the bias to begin encoding relational information early coexists with a second strong cross‐linguistic bias—the bias to encode agents early and to assign them to early sentence positions where possible. For example, an active, agent‐first sentence like “The thief stole the painting” is a more likely description of the event in the current example than a passive, patient‐first sentence like “The painting was stolen by the thief” in English as well as in numerous documented languages (see, e.g., Riesberg, Malcher, & Himmelmann, 2019). The agent‐first bias in word order reflects a conceptual hierarchy in event roles that is mirrored in an earlier attentional focus on agents than patients during event processing. Naturally, recognizing one event character as an agent requires encoding sufficient relational information about all characters to compare their conceptual features and identify their event roles. However, information about event roles is encoded rapidly during event viewing (Hafri, Papafragou, & Trueswell, 2013; Hafri, Trueswell, & Strickland, 2018; Isasi‐Isasmendi et al., 2023; also see Griffin & Bock, 2000, and Bock, Irwin, Davidson, & Levelt, 2003, for discussions of event apprehension), and agent‐like characters—by virtue of their function as “event‐builders” or prototypical “carriers” of relational information—attract attention in tasks involving both linguistic and nonlinguistic responses across languages (Cohn & Paczynski, 2013; Goldin‐Meadow et al., 2008; Isasi‐Isasmendi et al., 2023; Konopka, 2019; Nordlinger et al., 2022; Sauppe, 2017; Sauppe & Flecken, 2021; Sauppe et al., 2023; Webb, Knott, & MacAskill, 2010; also see Bornkessel‐Schlesewsky & Schlesewsky, 2009, and Rissman & Majid, 2019, for reviews). There is also a strong, albeit not deterministic, link between animacy and agenthood (e.g., Bock, Loebell, & Morey, 1992; Bock & Warren, 1985; Branigan, Pickering, & Tanaka, 2008; Grewe et al., 2006), so animate characters may attract attention as potential agents. In other words, agents have a more privileged relational role, so speakers may be more likely to build messages “around” agents (the “who” element in a who‐did‐what‐to‐whom framework) than “around” other event characters, like patients (the “to whom” element). This bias is consistent with linear word order in languages like English: earlier encoding of one character than another can result in something akin to a “left‐to‐right” bias at the message level, where characters that are conceptually more central to the event because of their contribution to generating a relational framework are allocated more early resources, while characters that are less central are produced later and can, therefore, be allocated fewer processing resources early in the planning process.

In sum, the order in which speakers encode message elements in a who‐did‐what‐to‐whom relational framework can be driven by the contribution that these elements make to generating this framework. In English, the joint effects of a bias to encode relational information and, in particular, a bias to prioritize encoding of agents early on are compatible with the canonical linear word order of transitive sentences (i.e., active SVO syntax). These biases can simplify the mapping between message‐level and sentence‐level information.

At the same time, questions about the breadth of message‐level planning remain. A “left‐to‐right” bias may be observed for different reasons: it may be observed systematically if it is indeed a preferred solution to the mapping problem (as described above for English), but it may also be observed as a consequence of production pressures and costs that a flexible incremental system tried to accommodate. Much like other cognitive processes, production processes are subject to central processing bottlenecks (e.g., Christiansen & Chater, 2016; MacDonald, 2013), and the size of planning increments can change to accommodate different processing requirements (e.g., Konopka, 2012; Konopka & Meyer, 2014; Konopka et al., 2018). For example, message‐level planning scope is reduced when speakers respond under time pressure (Ferreira & Swets, 2002) or under conditions of higher cognitive load (Wagner, Jescheniak, & Schriefers, 2010; also see Meyer, Sleiderink, & Levelt, 1998). In such cases, message‐level planning runs ahead of sentence‐level planning by a smaller margin than under lighter‐load conditions, resulting in a clear “left‐to‐right” bias. Importantly, it is also possible for a “left‐to‐right” bias to be observed as a result of *sentence‐level* constraints rather than message‐level constraints. Production requires outputting linearly ordered sequences of words; accordingly, sentence‐level planning must be consistent with linear word order and is, in fact, constrained by the ease of encoding individual words in that specific order. For example, speakers are able to plan the names of two objects in parallel if the first object is easy to encode but not when it is more difficult to encode (Griffin, 2003; Konopka, 2012; Meyer, Ouellet, & Hacker, 2008; Smith & Wheeldon, 1999; Wheeldon, Smith, & Apperly, 2013, 2011; see Roelofs & Piai, 2011, for a review). As the linear distance between two objects or characters increases, it is also more difficult to find evidence of properties of the second object influencing earlier processing. In such cases, it is possible that message‐level planning is, in principle, sufficiently extensive to incorporate information about multiple concepts, but that this extensive planning is quickly masked by immediate production costs, effectively resulting in a “left‐to‐right” bias that is nearly indistinguishable from that of a narrow‐scope, “left‐to‐right” message planning process.

Thus, new evidence is needed to assess the breadth of message planning scope during sentence production. This evidence may come from at least two approaches: (a) cross‐linguistic comparisons of the time‐course of planning for message elements that are produced in different sentence positions and (b) within‐language comparisons of the time‐course of planning for message elements with varying degrees of encoding difficulty.

## 2 Current study

This study took the latter approach and examined the breadth of message‐level planning by tracking the time‐course of planning of *patients*, that is, the “to whom” element in the relational who‐did‐what‐to‐whom message‐level framework that may be generated during production of transitive sentences with active syntax. The target language was English. Given the non‐privileged status of patients compared to agents and their late placement in active SVO sentences in English, speakers do not need to encode detailed patient‐specific information during early message planning in order to begin speaking. The extent to which they might nevertheless do so provides a stringent test of hypotheses about the upper bound of message‐level planning increments, or in other words, about the presence of “left‐to‐right” encoding biases during message planning.

**Fig. 1:**
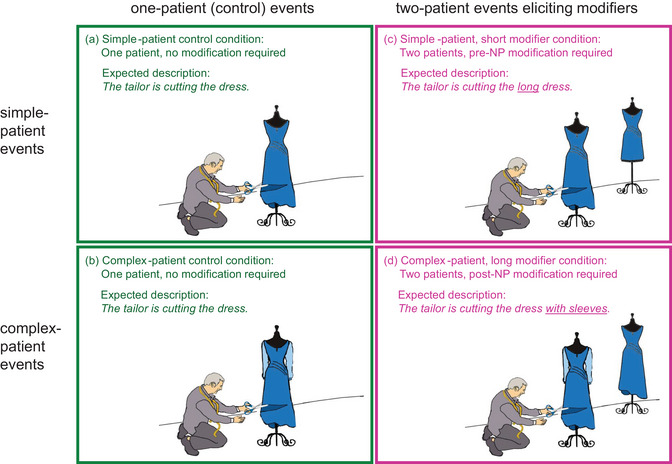
Schematic illustrating the 2×2 design of the experiment with examples of expected descriptions.

Sentences were elicited from pictures of simple events. To assess the amount of information that speakers might encode about patients, the target pictures were manipulated to elicit conceptually and linguistically light or heavy patient NPs by varying the number and complexity of the patient characters (Fig. 1). Two variables were manipulated: patient number and patient complexity. One‐patient events showed an agent acting on a single patient (conditions A and B) and elicited sentences where the target patient was described with an unmodified NP (e.g., “The tailor is cutting *the dress*”). Two‐patient events showed a non‐target patient standing next to the target patient, which required production of a modifier to uniquely identify the target patient (e.g., Brown‐Schmidt & Tanenhaus, 2006). Features of the target patient were manipulated to elicit shorter and longer modifiers (“The tailor is cutting *the long dress*” vs. “*the dress with sleeves*”; conditions C and D), so that differences in patient NP length across conditions reflected differences in the type of information needed to produce an informative modifier: production of short, prenominal modifiers required encoding of a simple conceptual feature that could be expressed linguistically with a single adjective (e.g., *long*), whereas production of longer, postnominal modifiers required encoding of a more complex feature that had to be expressed with a new noun in a postnominal adjectival phrase (e.g., *with sleeves*; see Brown‐Schmidt, 2009). Thus, the current design affords several comparisons testing the granularity of patient‐specific information encoded at different time points during production. Importantly, any changes in the descriptions of the target patients across conditions pertain to non‐relational properties of these patients: they have no bearing on the way speakers describe the agent characters (the “who” element) or the action performed by agents on patients (the “did what” element in the relational who‐did‐what‐to‐whom framework). Manipulating non‐relational properties of a sentence‐final character is essential, as speakers can, in principle, postpone planning of this character until they have encoded all the conceptual and linguistic information that is produced earlier in the sentence (the agent and the verb).

Additionally, several properties of English support a high degree of linearity in planning, which works against finding early effects of manipulations applied to sentence‐final characters. To assign agent and patient event roles, speakers need to encode only very basic relational information—that is, the fact that there is an agent and a patient and thus that speakers will need to produce a transitive sentence. A fixed word order allows speakers of English to plan SVO sentences by encoding all further information about agents (“who”), verbs (“did what”), and patients (“to whom”) in a predictable linear order. With a relatively simple case marking system that does not require overt marking of dependencies between event characters, speakers of English can begin planning the sentence‐initial agent character without a complete relational framework in place to guide or support encoding of the sentence‐final character. The sentence‐medial position of the verb also implies that speakers can postpone encoding of detailed relational information to be expressed with a verb (and thus any patient‐specific information that might influence selection of a verb) until after they have encoded the agent. Finally, because it is possible for speakers to incorporate new conceptual information into a developing message once speaking has already begun (e.g., Brown‐Schmidt & Konopka, 2008, 2015), the late placement of patients in SVO sentences gives speakers sufficient time to plan new patient‐specific information after speech onset and to still produce a fluent utterance. In other words, English grammar gives speakers little incentive to plan even rudimentary information about sentence‐final characters—that is, characters to be produced at a maximal linear distance from the sentence‐initial agent—early on. This is in contrast to languages with a larger repertoire of word orders and complex marking of dependencies, where grammatical requirements can call for earlier planning of information specific to both characters (e.g., see Norcliffe & Konopka, 2015, for a discussion).

To track planning, speakers’ eye movements and speech were recorded during the production task. Eye movements are goal‐driven, and a close relationship between gaze and planning processes has been systematically demonstrated across a range of production paradigms starting with Griffin and Bock (2000; also see Ferreira & Rehrig, 2019, for a discussion of linearization in eye movement research) and across numerous languages. While there is no consensus on a strict temporal separation between message‐level and sentence‐level encoding, earlier studies reported analyses carried out on agent fixations observed between 0 and 400 ms or between 200 and 600 ms to capture early message encoding processes (e.g., Hwang & Kaiser, 2014; Konopka & Meyer, 2014; Konopka, 2019; Konopka et al., 2018; Norcliffe et al., 2014; Nordlinger et al., 2022; Sauppe, Norcliffe, Konopka, van Valin, & Levinson, 2013; Sauppe, 2017). In studies eliciting SVO descriptions in languages with fixed word order like English, fixations to the two event characters show varying degrees of convergence in these time windows (Griffin & Bock, 2000; Konopka & Meyer, 2014). Eye movements observed after 600 ms are interpreted as indexing word‐by‐word sentence‐level planning. Specifically, after 400–600 ms, there is a sharp increase in agent‐direct fixations, indicating the onset of sentence‐level encoding of the agent (i.e., lexical retrieval of its name; Griffin, 2004). Agent‐directed fixations typically peak around 1000 ms and then begin to drop as speakers complete retrieval of the agent name and shift their attention to the patient before speech onset.

For current purposes, it is the distribution of *early* eye movements, that is, eye movements observed between picture onset and the peak of agent‐directed fixations, that provides key information about the extensiveness of early message‐level encoding (Gleitman et al., 2007; Griffin & Bock, 2000; Konopka & Meyer, 2014; Konopka et al., 2018). Linear and Hierarchical Incrementality predict a different distribution of early fixations, implying a different balance in the amount of time devoted to message‐level and sentence‐level encoding in this time window. A sharp early increase in fixations to *one* character after picture presentation (followed by sustained fixations on this character after 400–600 ms) indicates an early onset of sentence‐level encoding of that character, leaving less time for encoding of a broader message plan that includes information about both characters. In such cases, given the strong preference for agent‐first syntax in English, early message planning may be limited to encoding sufficient event information to identify the agent but need not entail encoding of any detailed information about the patient. In contrast, a lower likelihood of speakers fixating agents in this time window and a delayed rise in agent fixations after 400–600 ms implies that speakers spend more time encoding message‐level information about *both* characters, consistent with Hierarchical Incrementality, before beginning sentence‐level encoding of the agent. Thus, in the current study, the distribution of early fixations was compared across conditions to assess the extent to which speakers incorporated information about both characters in their message plans.

Manipulating properties of the patients was expected to result in early attention‐capture effects across conditions due to differences in the visual complexity of the pictured events. In one‐patient events, patients with complex features (a dress with sleeves; condition B) may attract more attention than patients with simple features (a dress without sleeves; condition A). Comparing one‐patient events (conditions A, B) to two‐patient events (conditions C, D) may also show early effects of character numerosity. These effects are expected under both Linear and Hierarchical Incrementality. Importantly, predictions about the extent to which early eye movements are modulated by *conceptual* and *linguistic* properties of the patients (such as the ease of encoding a patient name as well as the length of the patient NP) differ under the two accounts.

If speakers prefer to encode messages in small chunks, then early attention‐capture effects due to patient complexity should be short‐lived, and any conceptual and linguistic properties of a character that is produced in sentence‐final position should have little influence on early eye movements (*Hypothesis 1; narrow‐scope linear planning*). On this hypothesis, all further patient details may be encoded incrementally much later in the planning process—at a point when linguistic encoding for the agent and the sentence verb is completed and when the patient is the only message element left to encode.

In contrast, if early message planning involves generating a framework with information about both event characters, then early eye movements may be influenced by properties of the patient, such as the ease of identifying and naming this character and the need to produce a modifier. One possibility is that speakers encode only high‐level, coarse‐grained conceptual information about the patient at this stage (e.g., the fact that the patient is a dress and not a suit) but not specific patient features that require production of a modifier (*Hypothesis 2; broad‐scope*, *coarse‐grained message planning*). In this case, early eye movements may vary between one‐patient and two‐patient events (conditions A and B vs. conditions C and D), but eye movements in conditions eliciting short and long modifiers should not differ (conditions C vs. D). The early message plan may simply include a “tag” associated with the patient—a tag that flags up the presence of a contrast and thus the need to eventually encode finer‐grained detail about the target patient—but information needed to select an appropriate modifier may be encoded incrementally at a later point in time. A second possibility is that speakers do encode fine‐grained conceptual information about the patients early on, i. e., both high‐level information about the presence of a patient that requires modification and lower‐level information about the unique properties of the target patient to be expressed with a modifier (*Hypothesis 3; broad‐scope*, *fine‐grained message planning*). If so, then patients described with postnominal modifiers (condition D) should be fixated for more time than patients described with prenominal modifiers (condition C).

Comparisons across these conditions using measures that capture the difficulty of encoding individual patients across target items (name codability and length) provide a key test of the granularity of patient encoding. Specifically, the degree to which speakers access patient‐specific information should be reflected in interactions of the manipulated variables (Patient Number and Complexity) with the codability and length measures. Speakers should be more likely to fixate patients with longer names when they undertake encoding of that character; conversely, they may also use information about patient length (a preliminary assessment of the difficulty of encoding the patient name) to strategically direct attention away from patients and thereby temporally separate encoding of the two characters in early windows.

Importantly, in all cases, the effects of manipulations applied to sentence‐final patients on early eye movements must be interpreted within the context of variables that influence the production of sentence‐initial agents. By hypothesis, the production costs of the first‐mentioned character can constrain planning of other event information, creating a “left‐to‐right” bias. Observing an influence of the patient manipulations over and above effects of variables specific to the difficulty of encoding agent names (name codability and length) is strong evidence against a “left‐to‐right” bias in message planning.

For completeness, the early window analysis was followed up by analyses of eye movements observed post‐600 ms to evaluate the extent to which early planning priorities may continue to shape encoding during windows traditionally associated with sentence‐level planning. As the first character to be produced is the agent, one might expect the distribution of agent fixations observed after 600 ms and before speech onset to be modulated primarily by the ease of encoding the agent character (name‐related gazes; Griffin, 2004). If planning proceeds in a “left‐to‐right” manner, there should be little influence of the patient manipulations in this time window. In contrast, any effects of the patient manipulations would suggest strong message‐driven modulation of name‐related gazes.

Mirroring these predictions for eye movements, effects of patient number and patient complexity were also examined for speech onsets. Speech typically begins shortly after speakers have completed retrieval of agent names (∼2000 ms after picture onset) and can, therefore, be influenced by variables specific to the production of agent NPs but also variables influencing planning of subsequent material (Griffin, 2003; Malpass & Meyer, 2010; Meyer, Ouellet, & Häcker, 2008).

## 3 Method

### 3.1 Participants

Data were available from 51 participants (35 female, 16 male; aged 18–30), all native speakers of English and undergraduate students at the University of Aberdeen. Data from seven further participants were excluded due to low response rates and technical problems. All participants gave informed consent.

### 3.2 Materials and design

There were 64 target pictures showing transitive events in which an agent acted on a patient (e.g., a tailor cutting a dress; see Supplementary Appendix A). The pictures were modified from stimuli used in earlier production studies. All agents were easy‐to‐name animate characters, and the majority of the patients (43 out of 64) were inanimate to ensure high rates of production of SVO sentences.

The design crossed two factors, Patient Number and Patient Complexity, resulting in four versions of each event (Fig. 1). Pictures in conditions A and B showed one‐patient events, in which an agent acted on a single patient, and thus served as controls for conditions C and D. Conditions C and D showed two‐patient events, featuring an agent, a target patient, and a non‐target patient. The non‐target patient was a character or object of the same semantic category but was placed further away from the agent and was not acted upon. Only the target patient was to be mentioned, so two‐patient events required the production of modifiers for the target patient.

The target patients also varied in features that elicited NPs with prenominal and postnominal modifiers. In the simple one‐patient control condition (condition A), the patient could be described with a single noun (*the dress*). In the corresponding simple two‐patient condition eliciting short modifiers (condition C), the two patients differed in a feature that required the use of a prenominal modifier (a size or color adjective, e.g., *the long dress*) to uniquely identify the target patient. In the complex one‐patient control condition (condition B), the patient had an additional feature (sleeves) that did not require naming, as the event showed only one patient. In the corresponding complex two‐patient condition eliciting long modifiers (condition D), the two patients were distinguishable only by means of this feature, and thus speakers needed to produce an additional object name in a postnominal phrase (e.g., *the dress with sleeves*) to describe the target patient. These unique features were designed to be easy to spot (confirmed by the low incidence of descriptions that included nondiagnostic modifiers).

Target pictures were counterbalanced across four experimental lists, such that each item was presented in a different condition across lists, and, within lists, each item was seen in only one condition by each participant. Character placement in the pictures was counterbalanced: half of the target pictures were shown with the agent on the left and half with the agent on the right‐hand side of the picture. The target pictures were then interspersed among 106 fillers. Seven filler pictures served as practice trials at the beginning of the experiment, and all targets were separated by 1–2 fillers. The filler pictures showed a range of transitive and intransitive one‐character and multi‐character events. Multi‐character events showed groups of characters performing a single joint action (e.g., people waiting for a bus), and thus did not include character contrasts that required production of modifiers. In sum, the experimental lists included 138 pictures eliciting unmodified character NPs (32 one‐patient targets and 106 fillers) and 32 pictures eliciting modified patient NPs (32 two‐patient targets).

### 3.3 Procedure

Participants were informed they would see pictures of events with one, two, or more characters, and that their task was to produce one sentence to describe each event as quickly as possible in a way that would allow a potential listener to identify the characters shown in these events. Thus, in pictures with a single character, participants needed to describe the action performed by that character, while in pictures with two or more characters, they were asked to describe the characters that were interacting (target trials) or performing joint actions (filler trials). They were given one example of an event with two and three characters to familiarize them with the types of events they would see and to explain which characters were to be described.

### 3.4 Scoring

Trials where the first character fixation occurred later than 400 ms after picture onset were excluded. There were 2540 scorable sentences in total (2359 agent‐first sentences^4^, 130 passives, and 51 truncated passives). The remaining 724 sentences were rejected for a number of reasons (use of plural, generic, or mass nouns like “the washing,” preambles like “there is…,” indefinite pronouns like “someone,” and locatives like “the left apple”; mention of additional objects like “waving a stick at a bear”; perspective shifts or role reversals; early repairs and restarts). The proportions of scorable agent‐first sentences did not differ across conditions (assessed with a mixed‐effects logistic model, *z* = .995): agent‐first sentences made up 92% of all descriptions of one‐patient events and 93% of all descriptions of two‐patient events. Thus, any differences in early allocation of attention across conditions did not change speakers’ preference to start sentences with agents.

Given that the key manipulations concerned characters that were expected to be produced in sentence‐final position, only agent‐first sentences were considered for further analysis. A small number of sentences (80 in total) included unnecessary agent modifiers and were excluded. In the remaining dataset, trials with onsets longer than 3 standard deviations away from the overall mean were also excluded, leaving 2230 agent‐first sentences. These sentences were scored as including no patient modifier, a short modifier, or a long modifier (Table 1). Analyses were carried out on 1885 sentences with the expected modifier types (i.e., 85% of all scorable active sentences): 983 sentences with no modifiers describing one‐patient events and 902 sentences with the expected prenominal and postnominal modifiers describing two‐patient events.

**Table 1:** Number of sentences with and without modifiers in each cell of the 2×2 design

					Number of sentences with:					
Condition	Number of patients	Patient type	Expected modifier	Proportion of sentences with expected modifiers	No modifier	Short modifier		Long modifier		Other
						Pre‐NP	Post‐NP	Pre‐NP	Post‐NP	

(A) one‐patient events with simple patients (no modifiers)	One	Simple	None	90%	531	36	5	0	1	14
(B) one‐patient events with complex patients (no modifiers)	One	Complex	None	81%	452	13	0	7	67	16
(C) two‐patient events with simple patients, short modifiers	Two	Simple	Short (pre‐NP)	81%	48	478	50	2	1	11
(D) two‐patient events with complex patients, long modifiers	Two	Complex	Long (post‐NP)	85%	30	2	1	17	424	24

*Note*. Sentences where a modifier was required but none was provided (*n* = 78 in two‐patient events) were often followed by repairs (e.g., *The man kicked the fence… the bigger fence*), and these sentences were excluded from the analyses. “Other” responses included sentences with both a prenominal and postnominal modifier or with nondiagnostic modifiers. In the simple two‐patient events (condition C), the expected descriptions included prenominal modifiers (e.g., *…the younger man*) but speakers also used postnominal modification on a subset of trials to convey a similar meaning (e.g., *…the man with black hair*). Similarly, in the complex two‐patient events (condition D), the expected descriptions included postnominal modifiers (e.g., *…the fence with graffiti*) but speakers used prenominal modification on a subset of trials instead (e.g., *…the graffiti'ed fence*). Gray cells indicate responses with expected modifiers that were included in the analyses.

### 3.5 Character‐specific measures: Codability and NP length

To capture the variability associated with speakers’ use of different character names in their spontaneous productions, codability and NP length were calculated for all agents and patients.

Codability reflects the ease of conceptual and linguistic encoding, and is typically estimated with Shannon's entropy (Kuchinsky & Bock, 2010; Konopka & Meyer, 2014). High‐codability characters, or characters with high name agreement, are considered to be easy to identify and to name (e.g., *fireman*, *cat*). Characters with lower name agreement may be harder to identify (e.g., the words *bug*, *bee*, and *wasp* are all plausible names for an insect shown to be stinging a man's arm), indicating higher conceptual encoding difficulty, or may be referred to with a range of synonymous terms (e.g., the words *burglar*, *robber*, and *thief* are all plausible names for a person stealing a painting), indicating greater difficulty of linguistic selection (Kuchinsky et al., 2010). Analogous measures can be derived for verb codability from the range of verbs used in descriptions of each event (verb codability is reported for completeness but was not included in the analyses).

Here, codability scores were first calculated for agents, patients, and verbs from the distribution of scorable responses elicited in the eye‐tracked production task. To confirm the stability of these scores, an offline norming task was also conducted to obtain picture descriptions with a new sample of participants (50 native speakers of English recruited from Testable Minds; *M*_age_ = 25.3, *SD* = 3.67; 20 female, 29 male, 1 nonbinary). Participants received the same instructions as in the eye‐tracking task, but provided written descriptions (which removes the pressure to produce language quickly but fluently). The descriptions were scored using the same criteria as for the eye‐tracking task (with the exception of exclusions based on modifier placement), leaving 1984 responses for analysis. Descriptive statistics for codability scores from both tasks are listed in Table 2. Agent and Patient Codability scores were higher in the norming task than the eye‐tracking task, reflecting a wider range of character names (both *t*s(63)>3.5), but scores from both tasks were highly correlated (*r* = .72, .88, and .86 for Agent, Verb, and Patient Codability, respectively). On a reviewer's request, all analyses reported below used the codability scores from the offline task.

**Table 2:** Codability scores obtained in the offline norming task (written responses) and in the eye‐tracking task (verbal responses)

	Norming task		Eye‐tracking task	
	Mean (SD)	Range	Mean (SD)	Range
Agent codability	0.87 (0.61)	0–2.16	0.67 (0.62)	0–2.25
Verb codability	1.64 (0.94)	0–3.58	1.55 (1.00)	0–3.52
Patient codability	0.95 (0.74)	0–2.80	0.75 (0.70)	0–2.68

*Note*. Low values on the codability scale indicate higher name agreement and thus higher codability, while higher values indicate lower name agreement and thus lower codability. In both tasks, the codability scores for agents and patients did not differ, *t*s(63)<1. Agent and Patient codability were not correlated (all *r*s<.18).

Character NP lengths, including character name length and modifier length in syllables, were obtained for all scorable sentences in the eye‐tracked production task (Table 3). Character NP length arguably predicts the timing of lexical retrieval (see Damian, Bowers, Stadthagen‐Gonzalez, & Spalek, 2010, for a discussion), and in the current design, additionally reflects the complexity of the modifiers selected for two‐patient events. Agent character names did not differ in length across conditions (*t* = 1.96, *p* = .06, for the main effects of Patient Number, and *t* = 1.13, *ns*, for the main effect of Patient Complexity in a mixed‐effects analysis). As expected, there were differences in the length of patient NPs in descriptions of two‐patient events (*t*s>27 for the interaction of Patient Number and Patient Complexity), as, by design, these differences were driven by modifier type and length.

**Table 3:** Mean lengths (with standard deviations) of agent and patient NPs in syllables across conditions

	Agent NPs	Patient NPs		
Condition	Det + Agent	Det + Patient	Modifier	Total length
(A) one‐patient events with simple patients (no modifiers)	2.45 (.62)	2.68 (.83)	0	2.68 (.83)
(B) one‐patient events with complex patients (no modifiers)	2.50 (.68)	2.75 (.90)	0	2.75 (.90)
(C) two‐patient events with simple patients (short modifiers)	2.54 (.68)	2.77 (.86)	1.44 (.60)	4.21 (1.00)
(D) two‐patient events with complex patients (long modifiers)	2.55 (.69)	2.64 (.80)	4.90 (1.41)	7.53 (1.66)

### 3.6 Analyses

For the analysis of eye movements, three interest areas were defined for each pictured event: an agent area, a target patient area (which included any features relevant for producing a modifier), and a non‐target patient area. The analytical focus was on agent‐directed fixations, as agents were the first‐mentioned character. An early preference for fixating agents was expected based on linear word order under *Hypothesis 1*, and the patient manipulations were expected to modulate the magnitude of the agent preference under *Hypotheses 2* and *3*. For comparison against earlier work (e.g., Hwang & Kaiser, 2014; Konopka & Meyer, 2014; Konopka et al., 2018; Norcliffe et al., 2014; Nordlinger et al., 2022; Sauppe et al., 2013; Sauppe, 2017), analyses were carried out for three time windows of interest: (a) 200–600 ms; (b) 600–1800 ms; and (c) 1800–3000 ms. The 200–600 ms time window is argued to capture early eye movements reflecting message‐level encoding and the onset of sentence‐level encoding for the first‐mentioned character. The 600–1800 ms and 1800–3000 ms time windows (i.e., windows with eye movements observed before and after speech onset) are argued to primarily capture sentence‐level encoding of the agent and patient, respectively.

For these analyses, fixations were first binned into 10‐ms samples. To accommodate a large number of variables, agent‐directed fixations at the trial level were aggregated over time into the three analysis windows^5^ (see Fig. 2b−f) and submitted to mixed‐effects logistic regressions (Donnelly & Verkuilen, 2017) using the *lme4* package in R (R Core Team, 2024.12.0; Bates, Machler, Bolker, & Walker, 2015). The fixed factors were the manipulated variables (Patient Number, Patient Complexity), coded as (−.5, .5) for the two levels of each variable, as well as standardized continuous predictors (codability: Agent Codability, Patient Codability; length: Agent NP length, Patient NP length). The random effects structure of all models included random by‐participant and by‐item intercepts, as well as all random slopes that allowed convergence (listed in all tables).

Fig. 2Eye movements: by‐participant mean proportions of fixations (with confidence intervals) directed to agents, target patients, and non‐target patients (a) over a 4000‐ms time window (with dashed lines indicating speech onsets) and (b) averaged over the highlighted 200–600 ms, 600–1800 ms, and 1800–3000 ms time windows; model fits for the (c, d) three‐way interactions with Patient Length observed in each window; and model fits for the (e, f) three‐way interactions with Agent Length observed in each window, shown separately for simple‐patient and complex‐patient events.

**Figure:**
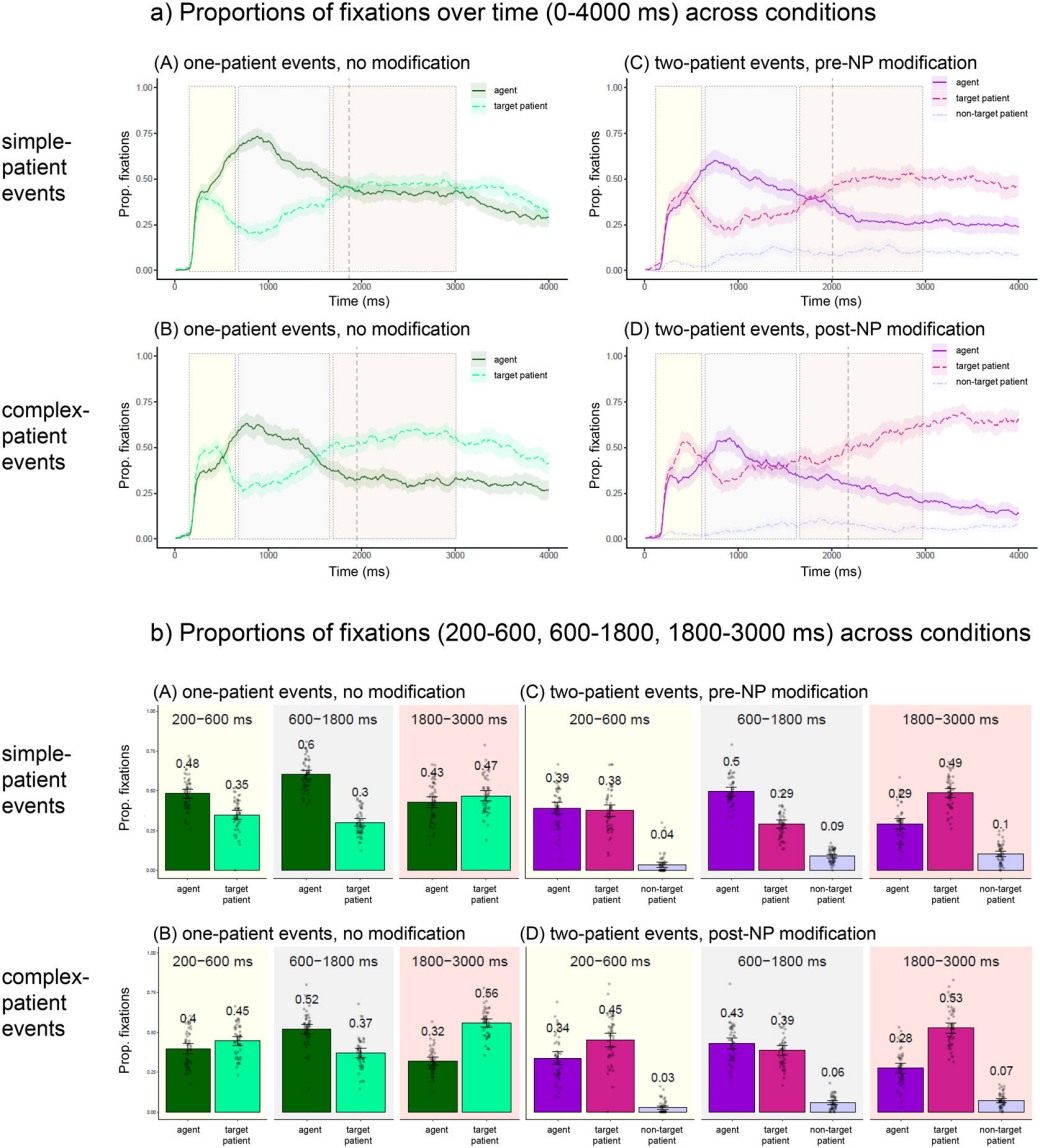

**Figure:**
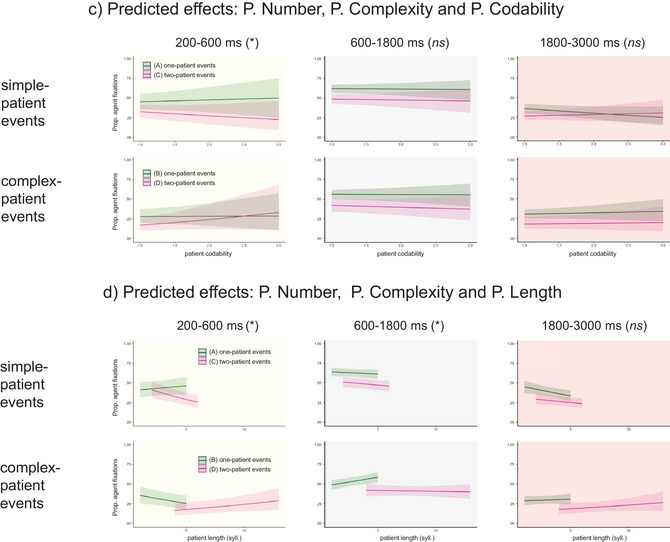

**Figure:**
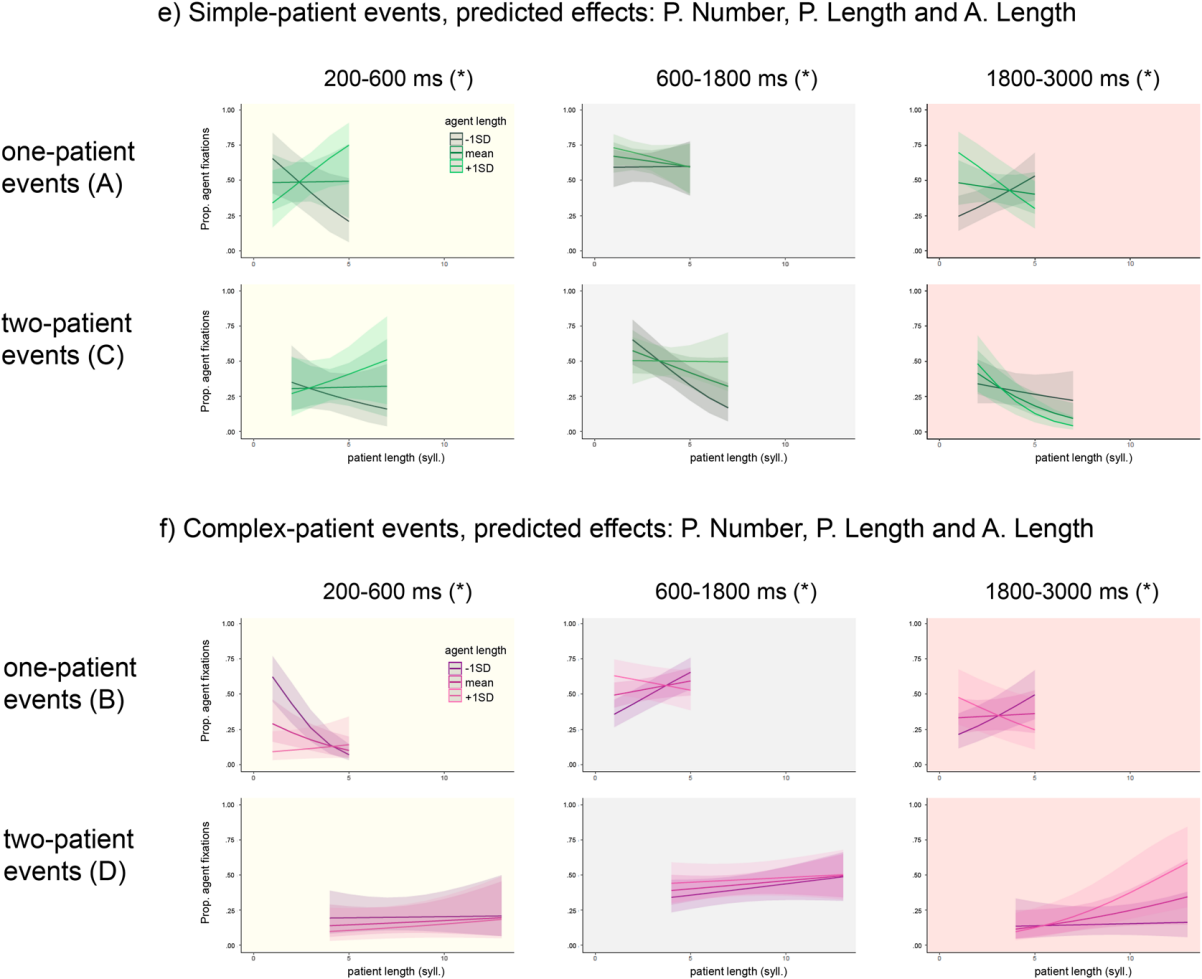

The analysis for each time window was carried out in two steps. In the *first* step, the models tested for all three‐way interactions between the two manipulated variables (Patient Number and Complexity) and the two patient‐specific predictors (Patient Codability and Length) in order to assess overall sensitivity to properties of the sentence‐final characters. For completeness, analogous analyses conducted on *patient‐directed* fixations are reported in Supplementary Materials. Modulation of these effects by properties of the sentence‐initial agent was assessed in the *second* step, in separate analyses conducted for descriptions of events with simple patients and complex patients. Comparing descriptions of events without modifiers (simple‐patient events: conditions A vs. C) and with modifiers (complex‐patient events: conditions B vs. D) tests whether and to what extent properties of sentence‐initial agents constrain planning of patients, separately for descriptions with short, prenominal modifiers and descriptions with long, postnominal modifiers. These analyses tested for all three‐way interactions between Patient Number and the codability and length variables (Patient Codability/Length and Agent Codability/Length). Only effects of interest observed consistently across all windows are described below (i.e., three‐way interactions between Patient Number, Patient Length, and Agent Length).

The final set of analyses compared speech onsets across conditions. As speech onsets are strongly influenced by properties of the sentence‐initial character, this analysis tested for all three‐way interactions between Patient Number, Patient Codability, and each of the continuous predictors (Agent Codability/Length, Patient Codability/Length) in one step.

All effects were considered significant at *p*<.05, determined with the *lmerTest* package (Kuznetsova, Brockhoff, & Christensen, 2017). All VIF values in the reported models were <5. Plots of predicted values were obtained with the *ggeffects* package (Lüdecke, 2018).

## 4 Results

### 4.1 Time‐course of planning

The distribution of fixations to three event characters (agents, target patients, and non‐target patients) across conditions is shown in Fig. 2a across a 4000‐ms time window beginning at trial onset. The first panel in Fig. 2a shows results for one‐patient events with simple patients (condition A). This condition is similar to earlier studies that used pictures of *two*‐character transitive events to elicit sentences requiring no modification (Hwang & Kaiser, 2014, 2015; Konopka & Meyer, 2014; Konopka et al., 2018; Konopka, 2019; Norcliffe et al., 2014; Nordlinger et al., 2022; Sauppe et al., 2013; Sauppe, 2017). Consistent with these studies, speakers began fixating the agent preferentially well within 600 ms of picture onset. Agent‐directed fixations then peaked around 1000 ms and began to decline in favor of a rise in patient‐directed fixations before speech onset. Crucially, in the current study, the likelihood of speakers fixating the agent over time differed across the remaining conditions (see the remaining three panels in Fig. 2a).

Mean proportions of fixations in the three windows are plotted separately for each condition in Fig. 2b, followed by plots of three‐way interactions with Patient Codability and Patient Length (Fig. 2c,d; see Table 4 for the full output).

### 4.2 Effects of patient‐specific predictors on planning

#### 4.2.1 All conditions: 200–600 ms time window (Table 4a)

As outlined in the Introduction, a rapid increase in agent‐directed fixations, and thus a higher proportion of fixations to agents, in the first time window (200–600 ms) is consistent with “left‐to‐right” planning: it suggests that speakers prioritize encoding of the agent. Conversely, a lower proportion of agent‐directed fixations in this time window suggests that speakers also devote resources to patients. Modulation of this pattern by the manipulated variables (Patient Number and Patient Complexity) as well as by production‐relevant properties of the patients (Patient Codability and Patient Length) was expected to demonstrate the degree of sensitivity to properties of the sentence‐final character during early planning.

**Table 4:** Results of logistic mixed‐effects regression analyses of agent‐directed fixations in the 200–600 ms, 600–1800 ms, and 1800–3000 ms time windows

	*b*	*SE*	*t*	*p*				*b*	*SE*	*t*	*p*				*b*	*SE*	*t*	*p*		
											
	(a) 200–600 ms							(b) 600–1800 ms							(c) 1800–3000 ms					
Fixed effects																				
Intercept	**−.89**	**.19**	**−4.79**	**<.0001**				.09	.09	.99	.32				**−1.00**	**.10**	**−9.93**	**<.0001**		
Patient Number	**−.60**	**.18**	**−3.29**	**<.001**				**−.57**	**.13**	**−4.29**	**<.0001**				**−.57**	**.19**	**−3.03**	**<.01**		
Patient Complexity	**−.84**	**.19**	**−4.29**	**<.0001**				**−.24**	**.11**	**−2.27**	**.02**				**−.38**	**.14**	**−2.65**	**<.01**		
Patient Codability	.06	.18	.32	.75				−.03	.08	−.42	.68				−.01	.09	−.06	.95		
Patient Length (syllables)	−.09	.09	−.99	.32				.004	.07	.05	.96				−.06	.10	−.59	.56		
Patient Number * Patient Complexity	−.07	.27	−.28	.78				−.04	.22	−.17	.86				−.25	.32	−.78	.44		
Patient Number * Patient Codability	.03	.11	.29	.77				−.04	.08	−.49	.62				.13	.12	1.10	.27		
Patient Number * Patient Length	−.03	.08	−.42	.68				**−.14**	**.04**	**−3.62**	**<.001**				**.09**	**.04**	**2.20**	**.03**		
Patient Complexity * Patient Codability	.23	.15	.155	.12				−.01	.07	−.21	.84				.12	.10	1.17	.24		
Patient Complexity * Patient Length	.09	.07	1.26	.21				**.19**	**.04**	**4.99**	**<.0001**				**.30**	**.04**	**7.42**	**<.0001**		
P. Number * P. Complexity * P. Codability	**.58**	**.19**	**3.07**	**<.01**				−.05	.14	−.34	.73				−.28	.21	−1.31	.19		
P. Number * P. Complexity * P. Length	**.96**	**.15**	**6.56**	**<.0001**				**−.19**	**.08**	**−2.55**	**.01**				−.04	.08	−.45	.65		
Random effects	Var.	SD	Corr.					Var.	SD	Corr.					Var.	SD	Corr.			
											
Items (intercept)	1.90	1.38						.37	.61						.39	.63				
Patient Number	.68	.82	.11					.37	.60	.11					.74	.86	.24			
Patient Complexity	1.27	1.13	.36	.24				.25	.50	.01	−.14				.54	.74	.14	.39		
P. Number * P. Complexity	1.62	1.27	.20	−.01	.37			1.00	1.00	−.06	.08	.35			2.43	1.56	.10	.29	.53	
Participants (intercept)	.18	.42						.09	.30						.18	.42				
Patient Number	.90	.95	.07					.54	.73	.05					1.15	1.07	.43			
Patient Complexity	.68	.82	.13	.61				.32	.56	.07	.72				.51	.72	.18	.60		
Patient Length	.27	.61	.00	−.82	−.72			.21	.45	−.05	−.92	−.65			.53	.73	−.17	−.82	−.66	
Patient Codability	.09	.29	.22	−.20	−.41	.17		.03	.18	.21	.19	.33	−.24		.08	.29	−.35	−.34	−.17	.28
P. Number * P. Complexity	1.39	1.18	−.12	.46	.58	−.57	−.29	1.49	1.22	.01	.66	.60	−.68	.16	2.79	1.67	.31	.71	.54	−.75 −.25

First, as expected, there was a main effect of Patient Complexity: speakers were less likely to fixate agents in events that featured complex patients (conditions B and D) than simple patients (conditions A and C), suggesting that early resources were devoted to patient characters that were visually more complex, irrespective of the need to produce a modifier (Fig. 2ab). Importantly, there was also a main effect of Patient Number: speakers were less likely to fixate agents in two‐patient events (conditions C and D) than in one‐patient events (conditions A and B), showing that more attentional resources were devoted to patients that required modification. Naturally, fixations to the target patient showed the inverse pattern. Fixations to the non‐target patient were very infrequent in this time window.

What type of patient‐specific information was encoded before 600 ms? The effects of Patient Number and Patient Complexity were modulated weakly by Patient Codability (Fig. 2c) and, to a larger extent, by Patient Length (Fig. 2d).

Fig. 2c shows an effect of Patient Codability mainly in two‐patient events. On trials requiring production of patients with short, prenominal modifiers (condition C), there were fewer fixations to agents (i.e., more fixations to patients) when patients were harder to name. This pattern reversed on trials requiring production of patients with long, postnominal modifiers (condition D): here, speakers spent more time fixating agents when patients were harder to name. Both effects show that speakers attempted to estimate the difficulty of encoding the patient early on but strategically elected to *postpone* encoding of this character when it required more complex modification (supporting *Hypothesis 2* but contrary to *Hypothesis 3*).

A similar effect was observed for Patient Length in two‐patient events (Fig. 2d). In sentences with short modifiers (condition C), there were fewer fixations to agents (i.e., more fixations to patients) as patient length increased, while in sentences with longer modifiers (condition D), speakers were more likely to maintain gaze on the agents as patient length increased. This demonstrates, again, strategic modulation of resources to postpone encoding of more complex patient information.

#### 4.2.2 All conditions: 600–1800 ms time window (Table 4b)

In earlier studies using stimuli corresponding to the simple one‐patient condition in the current design (condition A), eye movements observed between 600 ms and speech onset typically showed sustained fixations on the agent. In the current design, the likelihood of speakers fixating the agent was again modulated by Patient Number and Patient Complexity (Fig. 2a,b).

There were fewer agent fixations in events that featured complex patients (conditions B and D) than simple patients (conditions A and C; main effect of Patient Complexity), and fewer agent fixations in two‐patient events (conditions C and D) than one‐patient events (conditions A and B; main effect of Patient Number). This shows effects carrying over from the 200–600 ms time window, and the overall pattern provides a novel observation of how message‐level encoding priorities influence sentence‐level planning. One might expect that, if speakers spent more time fixating patients in some conditions than others before 600 ms, then this would delay the onset of agent‐directed fixations after 600 ms in those conditions, such that the distribution of agent‐directed fixations would shift “to the right” and that the length of time speakers spent fixating agents would stay constant across conditions (i.e., it would be sensitive to agent‐specific variables alone). Instead, Fig. 2a,b results show that there was a general decline in agent‐directed fixations, rather than a shift of the distributions to the right, after 600 ms in all the novel conditions (B, C, and D) relative to the simplest condition (A). In fact, the peak of agent‐directed fixations was still observed at around 1000 ms in all conditions. Thus, an early focus on patients consistently reduced the amount of time that speakers spent fixating agents even during a time window that is typically associated with priority sentence‐level encoding of the first‐mentioned character.

Eye movements in this time window were not influenced by Patient Codability (Fig. 2c) but showed some sensitivity to Patient Length (Fig. 2d): speakers were generally less likely to fixate agents when planning sentences with longer patient NPs, except in one condition (condition B, i.e., one‐patient events with complex patients).

#### 4.2.3 All conditions: 1800–3000 ms time window (Table 4c)

Eye movements in later windows tend to be noisier, but an analysis of the 1800–3000 ms window is reported for completeness. Speakers typically begin shifting their attention from agents to patients around or before speech onset. If message planning proceeded strictly “from left to right,” effects of the manipulated variables (Patient Number and Patient Complexity) would only be observed at this stage, that is, the latest point in time when speakers must begin encoding the sentence‐final patient. The analyses of previous time windows ruled out this possibility, so unsurprisingly, the analysis of the current window showed persistent effects of Patient Number and Patient Complexity (Fig. 2a,b). Interactions with Patient Codability were not significant (Fig. 2c), but Patient Length entered into two two‐way interactions with Patient Number and Patient Complexity (with the latter being a stronger effect; Fig. 2d). Speakers were more likely to have already shifted their attention to the patient character in two‐patient than in one‐patient events and in complex‐patient than in simple‐patient events, so modulation of eye movements by Patient Length was primarily observed in conditions where speakers were still fixating agents at the beginning of this window. In both one‐patient events and in simple patient events, there were fewer fixations to agents in sentences with longer patient names.

#### 4.2.4 Summary across windows

Comparing across time windows, the pattern of effects observed with the manipulated variables (Patient Number and Complexity) as well as with Patient Length suggests early sensitivity to the patient manipulations as well as linguistic properties of individual patients. The direction of these effects shows that speakers were generally more likely to allocate resources to patients in the conditions that placed higher processing demands (conditions B−D) compared to the simplest condition (condition A), but that their processing of individual patients did not involve encoding of fine‐grained details, supporting *Hypothesis 2*.

### 4.3 Effects of agent‐specific predictors on planning

Follow‐up analyses were carried out with the inclusion of agent‐specific variables to assess the extent to which properties of an earlier‐mentioned character influenced the processing of the sentence‐final patient. If speakers prioritize encoding of event characters “from left to right,” then properties of the sentence‐initial agent should limit the influence of patient‐specific variables on early eye movements. These analyses were carried out separately for events with simple patients (Fig. 2e) and complex patients (Fig. 2f) to compare the effects of agent‐specific variables in sentences with and without short modifiers (conditions A and C) and sentences with and without long modifiers (conditions B and D). The analyses tested for three‐way interactions between Patient Number, Patient Codability, and the two agent‐specific variables individually (Agent Codability and Length). Removing the agent‐specific variables from any model resulted in significantly poorer model fits (all *p*s<.0001).

All analyses showed interactions between Patient Number, Patient Length, and Agent Length, and are described in detail below. Interactions with Agent Codability were observed only in three analyses and are shown in Fig. S2.

**Table 5:** Results of logistic mixed‐effects regression analyses of agent‐directed fixations in the 200–600 ms, 600–1800 ms, and 1800–3000 ms time windows, separately for simple‐patient and complex‐patient events

	*b*	*SE*	*t*	*p*				*b*	*SE*	*t*	*p*				*b*	*SE*	*t*	*p*			
												
SIMPLE PATIENT EVENTS	(a) 200–600 ms							(b) 600–1800 ms							(c) 1800–3000 ms						
Fixed effects																					
Intercept	−.40	.32	−1.24	.21				−.17	.20	.82	.41				**−.75**	**.21**	**−3.48**	**<.001**			
Patient Number	**−.77**	**.39**	**−1.99**	**<.05**				**−.60**	**.27**	**−2.23**	**.03**				**−.82**	**.26**	**−3.16**	**<.01**			
Agent Codability	−.17	.31	−.53	.59				.19	.19	.99	.32				.02	.18	.13	.89			
Agent Length (syllables)	**.57**	**.21**	**2.65**	**<.01**				.11	.12	.92	.36				−.21	.22	−.97	.33			
Patient Codability	−.33	.25	−1.34	.18				−.28	.15	−1.88	.06				−.36	.20	−1.85	.06			
Patient Length (syllables)	.06	.46	.13	.90				−.33	.30	−1.11	.27				**−.54**	**.28**	**−1.96**	**.05**			
Patient Number * Agent Codability	−.37	.32	−1.17	.24				.22	.22	1.02	.31				**.72**	**.21**	**3.44**	**<.001**			
Patient Number * Agent Length	**−.58**	**.17**	**−3.44**	**<.001**				.11	.09	1.29	.20				−.06	.08	−.78	.43			
Patient Number * Patient Codability	−.36	.20	−1.80	.07				.15	.13	1.16	.25				.28	.20	1.37	.17			
Patient Number * Patient Length	.01	.17	.04	.97				**−.26**	**.08**	**−3.08**	**<.01**				**−.63**	**.08**	**−7.93**	**<.0001**			
Agent Codability * Patient Codability	.30	.23	1.33	.18				−.14	.14	−.99	.32				−.00	.14	−.01	.99			
Agent Codability * Patient Length	.42	.43	.99	.32				−.20	.26	−80	.43				**−.34**	**.05**	**−7.10**	**<.0001**			
Agent Length * Patient Codability	**−.30**	**.17**	**−2.33**	**.02**				.04	.10	.42	.67				−.08	.18	−.44	.66			
Agent Length * Patient Length	**.76**	**.17**	**4.36**	**<.0001**				.16	.09	1.65	.10				**−.69**	**.05**	**−14.89**	**<.0001**			
P. Number * P. Codability * A. Codability	.33	.19	1.69	.09				.01	.13	.05	.96				.21	.20	1.04	.30			
P. Number * P. Codability * A. Length	.07	.12	.61	.54				**.34**	**.06**	**5.80**	**<.0001**				.02	.07	.35	.73			
P. Number * P. Length * A. Codability	.04	.20	.21	.83				**.33**	**.10**	**3.27**	**<.01**				**.71**	**.09**	**7.52**	**<.0001**			
P. Number * P. Length * A. Length	**−.59**	**.17**	**−3.46**	**<.001**				**.68**	**.08**	**8.79**	**<.0001**				**.27**	**.08**	**3.20**	**<.01**			
Random effects	Var.	SD	Corr.					Var.	SD	Corr.					Var.	SD	Corr.				
												
Items (intercept)	4.35	2.09						1.68	1.30						1.10	1.05					
Patient Number	4.51	2.12	−.55					2.25	1.50	−.37					2.11	1.45	.31				
Agent Length	1.02	1.01	−.47	.42				.35	.59	−.38	.05			–	1.36	1.17	.33	−.17			
Patient Length	8.14	2.85	.62	−.79	−.35			3.05	1.75	.62	−.81	−.25	–								
Participants (intercept)	.86	.92						.41	.64						.85	.92					
Patient Number	.2.44	1.56	−.23					1.40	1.18	−.66					1.27	1.13	−.51				
Agent Codability	.30	.55	.11	−.06				.12	.34	−.01	.19				.52	.72	.13	.08			
Agent Length	.47	.69	.22	.11	−.42			.18	.42	.16	−.27	−.32			.55	.74	.17	.06	.05		
Patient Codability	.24	.49	.48	.04	−.13	−.06		.10	.32	−.05	.15	−.25	.05		.57	.76	−.17	−.15	−.44	−.31	
Patient Length	2.25	1.50	.70	−.64	−.08	.18	.17	1.35	1.16	.74	−.83	−.20	.18	−.11	3.76	1.94	.66	−.63	−.09	−.13	.29
COMPLEX PATIENT EVENTS	(d) 200‐600 ms							(e) 600‐1800 m							(f) 1800‐3000 ms						
Fixed effects																					
Intercept	**−1.88**	**.33**	**−5.75**	**<.0001**				−.04	.14	−.29	.77				**−1.27**	**.24**	**−5.37**	**<.0001**			
Patient Number	.06	.51	.11	.91				**−.78**	**.30**	**−2.60**	**<.01**				**−1.41**	**.62**	**−2.28**	**.02**			
Agent Codability	−.12	.25	−.47	.64				**.29**	**.12**	**2.34**	**.02**				.23	.20	1.15	.25			
Agent Length (syllables)	−.18	.26	−.71	.48				.05	.16	.33	.74				−.23	.26	−.89	.38			
Patient Codability	−.03	.32	−.10	.92				−.11	.14	−.83	.41				.14	.22	.64	.52			
Patient Length (syllables)	−.26	.21	−1.23	.22				.19	.12	1.67	.10				.19	.21	.92	.36			
Patient Number * Agent Codability	**−.56**	**.28**	**−2.02**	**.04**				**−.40**	**.14**	**−2.84**	**<.01**				−.04	.28	−.15	.88			
Patient Number * Agent Length	**−.40**	**.17**	**−2.33**	**.02**				**.30**	**.08**	**3.89**	**<.0001**				.14	.09	1.46	.15			
Patient Number * Patient Codability	.34	.23	1.45	.15				−.05	.13	−.40	.73				.18	.28	.65	.52			
Patient Number * Patient Length	**.80**	**.15**	**5.42**	**<.0001**				**−.13**	**.07**	**−1.99**	**<.05**				**.29**	**.08**	**3.68**	**<.001**			
Agent Codability * Patient Codability	.35	.22	1.60	.11				−.07	.11	−.68	.50				−.16	.16	−.98	.33			
Agent Codability * Patient Length	**.40**	**.09**	**4.74**	**<.0001**				**.28**	**.04**	**7.38**	**<.0001**				**−.11**	**.04**	**−2.50**	**.01**			
Agent Length * Patient Codability	**−.56**	**.22**	**−2.48**	**.01**				−.08	.15	−.54	.59				−.18	.22	−.84	.40			
Agent Length * Patient Length	**.51**	**.07**	**6.83**	**<.0001**				**−.24**	**.03**	**−7.79**	**<.0001**				**−.17**	**.04**	**−4.29**	**<.0001**			
P. Number * P. Codability * A. Codability	−.22	.23	−.96	.34				−.20	.13	−1.51	.13				.31	.27	1.15	.25			
P. Number * P. Codability * A. Length	**−.87**	**.13**	**−6.57**	**<.0001**				**.43**	**.06**	**7.13**	**<.0001**				.10	.08	1.35	.18			
P. Number * P. Length * A. Codability	−.03	.17	−.16	.87				**−.16**	**.08**	**−2.08**	**.04**				.08	.09	.93	.35			
P. Number * P. Length * A. Length	**−.86**	**.15**	**−.587**	**<.0001**				**.39**	**.07**	**6.00**	**<.0001**				**.88**	**.08**	**10.71**	**<.0001**			
Random effects	Var.	SD	Corr.					Var.	SD	Corr.					Var.	SD	Corr.				
												
Items (intercept)	4.56	2.14						.48	.69						1.09	1.04					
Patient Number	2.28	1.51	.03					.80	.90	−.14					3.71	1.93	−.03				
Agent Length	1.54	1.24	.74	−.18				.88	.94	.25	−.14				1.82	1.35	.25	.01			
Participants (intercept)	.87	.93						.31	.56						1.22	1.10					
Patient Number	9.64	3.10	.62					3.55	1.88	.47					15.64	3.95	.76				
Agent Codability	.40	.64	−.10	−.24				.11	.34	.30	.36				.53	.73	.28	.21			
Agent Length	.93	.97	−.10	−.10	−.05			.22	.47	.11	.08	−.13			1.19	1.09	−.30	−.49	−.40		
Patient Codability	.61	.78	.02	−.19	−.08	−.34		.21	.45	−.08	−.40	−.23	−.17		.75	.87	−.39	−.44	−.23	.27	
Patient Length	1.89	1.38	−.59	−.92	.23	−.07	.13	.61	.78	−.50	−.92	−.36	−.12	.43	2.13	1.46	−.63	−.90	−.01	.37	.22

#### 4.3.1 Simple‐patient events: 200–600 ms time window (Table 5a)

In simple‐patient events, there was a trade‐off between fixating agents and patients with shorter and longer NPs. When the patient name was long, speakers allocated attention to the patient character only when the sentence‐initial agent NP was short and thus easy to produce. When the sentence‐initial agent NP was longer, speakers were more likely to continue fixating the agent and thus postpone encoding of the patient. In other words, speakers were sensitive to properties of both characters, but the length of the agent name determined strategic allocation of attention to the patient. This effect was stronger in sentences that did not require patient modifiers (one‐patient events, i.e., condition A) than in sentences with modifiers (two‐patient events, i.e., condition C).

#### 4.3.2 Simple‐patient events: 600–1800 ms time window (Table 5b)

In the time window associated with sentence‐level encoding of the first‐mentioned character, eye movements were expected to reflect the amount of time needed to encode that character. Consistent with this expectation, in simple‐patient events requiring no modification, speakers were more likely to fixate agents described with longer NPs (condition A). Planning of two‐patient events (condition C) showed the same trade‐off between agent and patient fixations as that observed in the 200–600 ms window, with speakers being more likely to fixate agents with longer names if patients also had longer names. This demonstrates a strategic focus on encoding of the agent character. When agent names were shorter, there were more fixations to the patient, modulated by patient name length.

#### 4.3.3 Simple‐patient events: 1800–3000 ms time window (Table 5c)

As attention generally shifted to patients after speech onset, all sentences showed the opposite trade‐off to that observed in the 200–600 ms time window for agent and patient length. Speakers were more likely to still fixate agents with longer names only when patient NPs were short, but as patient length increased, the shift to fixating the patient was more pronounced (particularly in two‐patient events). This shift was likely facilitated by the increased focus on agents with longer names in the previous window (in sentences with modifiers; condition C), which successfully reduced the amount of time that needed to be devoted to these agents after speech onset.

Comparing these effects across time windows shows the persistence of a clear trade‐off between attending to agents and patients of different lengths over time. In earlier time windows, speakers generally avoided attending to patients with longer names if the agent NP was also long. Early preferential encoding of these agents then allowed more time to be allocated to patients in later windows. This pattern is consistent with a “left‐to‐right” encoding strategy where the two characters are encoded sequentially.

#### 4.3.4 Complex‐patient events: 200–600 ms time window (Table 5d)

In complex‐patient events, a pattern of sensitivity to Agent Length resembling the trade‐off observed in simple‐patient events was present only when patient modification was not required (i.e., in condition B). There were more agent fixations in sentences with short agent NPs, and the likelihood of speakers fixating the agent dropped when patient names were longer. Fixations on agents described with longer NPs were less frequent. When describing events that elicited the longest and most complex patient descriptions (condition D), speakers were generally less likely to fixate agents overall, and the effects of agent NP length were weak.

#### 4.3.5 Complex‐patient events: 600–1800 ms time window (Table 5e)

A trade‐off similar to that observed in simple‐patient events with no modifiers (condition A) was present in complex‐patient events with no modifiers (condition C). Speakers fixated agents described with longer NPs when patient NPs were short, but were more likely to shift their attention to patients when patient length increased. In two‐patient events (condition D), there was again less sensitivity to agent NP length.

#### 4.3.6 Complex‐patient events: 1800–3000 ms time window (Table 5f)

Finally, a trade‐off was again observed in the last window in complex‐patient events without modifiers (condition B). There was little influence of Agent Length in descriptions with modifiers (condition D), with a surprising reversal in sentences with the longest patient NPs (to be interpreted with caution).

Overall, effects of Agent Length were observed primarily in complex‐patient events without modifiers (condition C) and were generally consistent with those of descriptions produced for simple‐patient events without modifiers (condition A). Departures from this pattern were observed in the most complex condition requiring production of longer modifiers (condition D). Here, there were more patient fixations overall, reflecting the strong effect of the Patient Number manipulation, but the likelihood of speakers fixating agents before speech onset increased when patients were described with longer NPs. This pattern was not further modulated by Agent Length. In other words, eye movements in this condition were strongly influenced by high‐level properties of the events and production of sentence‐final heavy NPs.

### 4.4 Speech onsets

**Fig. 3:**
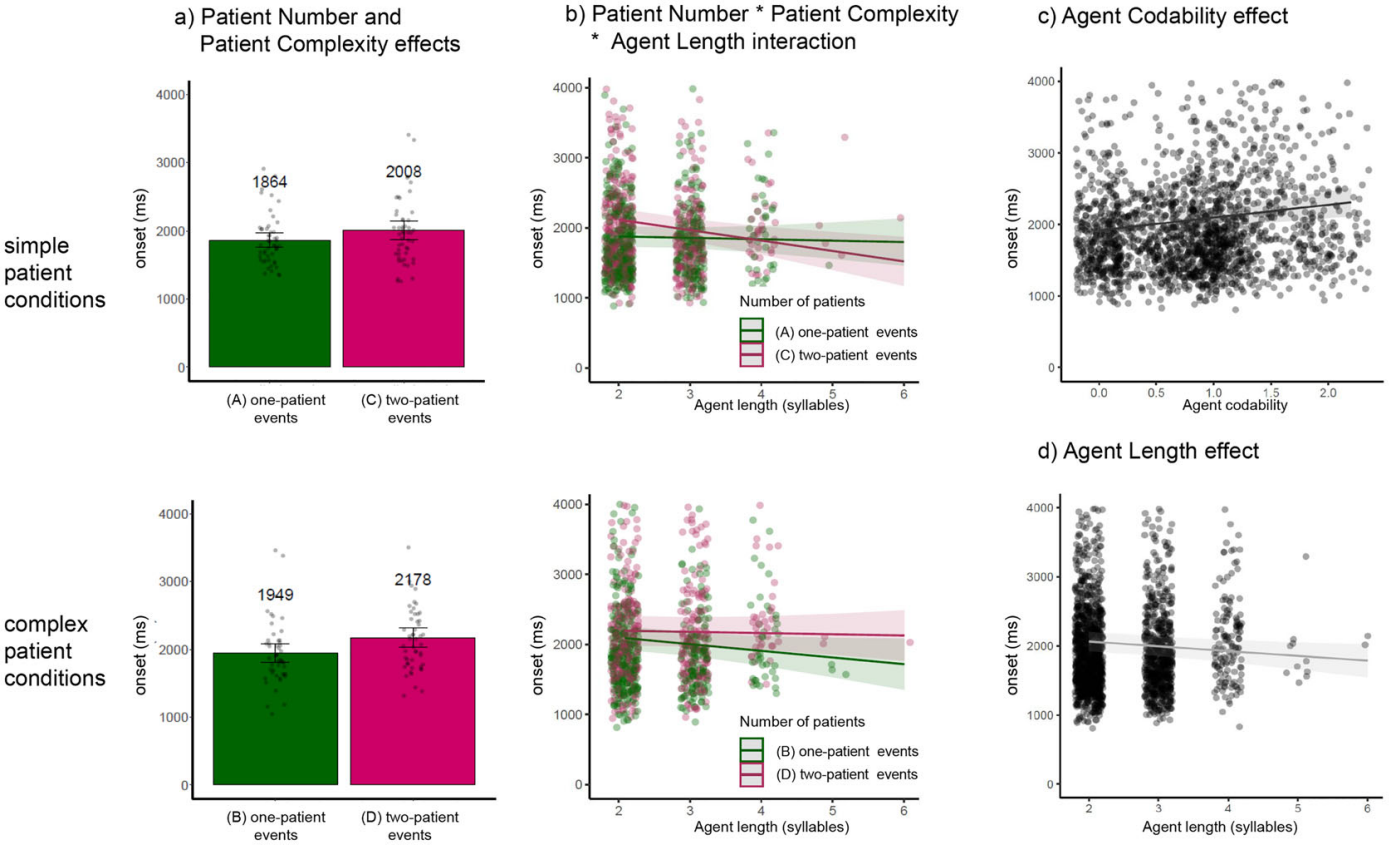
Speech onsets: (a) by‐participant means across conditions, and data and predicted effects for (b) the interaction between Patient Number, Patient Complexity, and Agent Length, (c) the main effect of Agent Codability, and (d) the main effect of Agent Length.

Speech onsets occurred approximately 2000 ms after trial onset (i.e., at the start of the third analysis window for eye movements) and were thus expected to vary with the manipulation of Patient Number and Patient Complexity, as well as to show sensitivity to properties of the agent character about to be named. There were two noteworthy results (Table 6 and Fig. 3).

**Table 6:** Results of the linear mixed‐effects analyses of speech onsets

	*b*	*SE*	*t*	*p*	
Fixed effects					
**Intercept**	**2039**	**70**	**29.18**	**<.0001**	
**Patient Number**	**156**	**64**	**2.44**	**.02**	
**Patient Complexity**	**150**	**55**	**2.72**	**<.01**	
**Agent Codability**	**109**	**32**	**3.37**	**<.01**	
Patient Codability	13	32	.42	.68	
**Agent Length**	**−46**	**19**	**−2.40**	**.02**	
Patient Length	28	37	.76	.45	
Patient Number * Patient Complexity	−39	101	−.38	.70	
Patient Number * Agent Codability	−10	30	−.34	.74	
Patient Number * Agent Length	−17	27	−.65	.52	
Patient Number * Patient Codability	43	30	1.43	.16	
Patient Number * Patient Length	−23	61	−.38	.71	
Patient Complexity * Agent Codability	−26	33	−.79	.44	
Patient Complexity * Agent Length	19	29	.68	.50	
Patient Complexity * Patient Codability	6	34	.18	.86	
Patient Complexity * Patient Length	50	59	.85	.40	
P. Number * P. Complexity * A. Codab.	−36	61	−.59	.56	
P. Number * P. Complexity * A. Length	**136**	**55**	**2.49**	**<.02**	
P. Number * P. Complexity * P. Codab.	63	61	1.04	.30	
P. Number * P. Complexity * P. Length	−74	113	−.65	.51	
Random effects	Variance	SD	Corr.		
Items (intercept)	52341	229			
Patient Number	18177	134	−.19		
Patient Complexity	29364	171	.08	−.32	
P. Number * P. Complexity	74130	272	−.27	.08	.11
Participants (intercept)	175331	419			
Patient Number	51067	226	.75		
Patient Complexity	17643	133	.67	.84	
Patient Length	10409	102	−.42	−.66	−.79
Residual	209818	458			

First, both Patient Number and Patient Complexity predicted the onset of articulation (Fig. 3a). Speech onsets were longer in conditions with complex patients (conditions B and D) than in conditions with simple patients (conditions A and C; main effect of Patient Complexity). This effect was independent of Patient Number, suggesting that part of the onset difference between sentences describing events with simple and complex patients can be attributed to delays due to processing of visually more complex patients, irrespective of modifier production. However, speech onsets were also longer in descriptions of two‐patient events (conditions C and D requiring modification) than one‐patient events (conditions A and B not requiring modification; main effect of Patient Number), suggesting an effect of the conceptual and linguistic complexity of the patient characters on the onset of articulation. These effects mirror the results of the eye movement analyses in the two pre‐speech (200–600 and 600–1800 ms) time windows.

Second, speech onsets were further modulated by production‐relevant properties of the first‐mentioned character, that is, Agent Codability and Agent Length. Agent Codability captures the conceptual and linguistic difficulty of selecting an agent name; accordingly, onsets were shorter in sentences with easier‐to‐name than harder‐to‐name agents (main effect of Agent Codability; Fig. 3c). In contrast, Agent Length captures the lower‐level difficulty of retrieving a phonological form and generating an articulatory plan. Onsets were generally *longer* in sentences with shorter agent NPs and *shorter* in sentences with longer agent NPs (main effect of Agent Length; Fig. 3d). This reversed word length effect (Griffin, 2003; Meyer, Belke, Häcker, & Mortensen, 2007) suggests strategic regulation of the onset of articulation: onsets were delayed when agent NPs were short because speakers were able to start planning other material in parallel.

The interaction of Agent Length with the two manipulated variables (Patient Number and Patient Length; Fig. 3b) shows different effects of the length of the sentence‐initial NP on speech onsets primarily in two‐patient events where modification was required (conditions C and D). In two‐patient events eliciting short modifiers (condition C), onsets showed the reversed length effect described above: longer onsets when agent NPs were shorter (consistent with more fixations being directed to patients) and shorter onsets when agent NPs were longer (consistent with fewer fixations being directed to patients). This effect was not present in the condition eliciting the most complex patient descriptions (condition D): here, agent NP length did not modulate speech onsets (Fig. 3b). This finding is consistent with the weakness of agent NP length effects in eye movements in the pre‐speech time windows, and suggests that allocating more attention to patients in this condition overall resulted in much weaker modulation of speech onsets by agent‐specific properties.

## 5 Discussion

This study assessed the breadth of message‐level planning scope during the production of descriptions of two‐character transitive events. How extensive are the message plans that speakers generate at the outset of production? The amount of detail encoded about different elements of a message is difficult to gauge, partially because production pressures can result in speakers prioritizing encoding of information “from left to right,” that is, in an order that is consistent with linear word order. Here, manipulating properties of the *sentence‐final* patient character and assessing sensitivity to these properties in early eye movements was expected to show whether early message planning extended to this character, and if so, what type of information about this character was encoded in the early message plan. Three aspects of the design provide a strong test of the hypotheses: the sentence‐final placement of patients in SVO sentences, the manipulation of non‐relational properties of the patients, and the lack of grammatical dependencies in the target language (English) that would explicitly require early planning of patient‐specific information.

### 5.1 Summary of eye movement results

Analyses of early eye movements (200–600 ms) showed several new findings. First, speakers did not prioritize encoding of the sentence‐initial agent character to the same extent across conditions (contrary to *Hypothesis 1: narrow scope linear planning*). Instead, they also allocated attention to the sentence‐final patient character, and the degree to which they did so was modulated by lower‐level perceptual factors as well as by production‐relevant variables. As expected, the number of patient characters (one vs. two) as well as differences in the visual and conceptual complexity of the target patient (simple vs. complex) modulated allocation of attention to the patients. Importantly, there was also evidence of early sensitivity to *production‐relevant* properties of both patients and agents (codability and length). The strongest effects of patient codability and length were observed in two‐patient conditions, where speakers were more likely to maintain gaze on agents when their descriptions would include patients that were harder to name (codability) and that elicited longer NPs (length). Given that it is unlikely that speakers began linguistic encoding of patient names in this early time window, these effects suggest that speakers engaged in a process of estimating the encoding complexity of patient characters and, to some extent, estimating the difficulty of encoding modifiers as well. Importantly, the direction of both effects shows that speakers tried to *postpone* encoding of finer‐grained patient‐specific details when patients would require more processing resources (supporting *Hypothesis 2: broad‐scope*, *coarse‐grained planning*, but contrary to *Hypothesis 3: broad‐scope*, *fine‐grained planning*).

Analyses of eye movements in later windows (before and after speech onset: 600–1800 ms and 1800–3000 ms) were carried out to test for effects of the patient manipulations on sentence‐level encoding. There were large differences in the likelihood of speakers fixating agents across conditions that were driven by the complexity of the to‐be‐described events. This was the case even though the descriptions of the agent characters themselves did not change across conditions. In other words, while speakers had to devote resources to preparing the agent NPs first, the relationship between eye movements and lexical retrieval of the sentence‐initial character was substantially weaker than observed previously. There was tight coordination between eye movements and encoding of the agent name only in the simplest condition (replicating results shown starting with Griffin & Bock, 2000), while in conditions with more complex patient characters, speakers looked at agents for less time and began shifting their attention to the more resource‐demanding task of encoding patient characters in the 600–1800 ms time window. Thus, instead of reflecting the cost of sentence‐level encoding alone, eye movements showed strong anticipatory effects of the high‐level complexity of the sentence‐final character. In the current context, these effects can be attributed to information encoded at the message level from the earlier stages of planning, and are a novel example of goal‐driven shifts of visual attention in sentence production.

Interactions with agent‐specific variables in these windows showed continuous sensitivity to the length of agent and patient NPs. Effects of an agent‐specific variable are, of course, expected given that sentence‐level encoding proceeds “from left to right” and that character fixations index the difficulty of encoding that character's name (name‐related gazes). Importantly, the trade‐offs observed between agent and patient NP length on the likelihood of fixating the agent generally show a preference for maintaining gaze on agents before speech onset when patient NPs were longer, i. e., an attempt to temporally separate encoding of agents and patients in sentences with longer sentence‐final NPs. Thus, sensitivity to properties of the patients had to be squared with the need to produce a different character name in the sentence‐initial subject position first. The condition with the lowest degree of early modulation of eye movements by agent length was the condition with the longest patient names. It is likely that increased attention to complex patients reduced speakers’ reliance on agent‐specific information to strategically allocate resources.

### 5.2 Summary of speech onset results

Speech onsets were sensitive to the manipulated variables as well as properties of the sentence‐initial character. This is expected given that articulation started approximately 2 s into each trial, by which time speakers had generated messages that included conceptual information about both characters and had encoded the sentence‐initial NP.

The agent codability and length variables had different effects, in keeping with their hypothesized relationship to character naming. Sentences with high‐codability agents had shorter onsets than sentences with low‐codability agents. Once a suitable lexical item is retrieved, its length can influence the speed of phonological encoding and thus the onset of articulation. Replicating earlier findings, there was evidence of strategic regulation of the timing of articulation by the length variable: onsets were delayed when the agent NP was short and thus easy to produce. This trade‐off between planning an agent name and planning material beyond the agent at speech onset is consistent with “left‐to‐right” encoding priorities. When material on the “left” is easy to encode, speakers can start to plan additional material in parallel; when material on the “left” is more difficult to encode, less material on the “right” can be included in the same planning window (Griffin, 2003; Konopka, 2012; Meyer et al., 2008). Relatedly, when the material on the “right” is harder to encode, speech onsets are shorter as the planning scope is restricted to one character or object (Malpass & Meyer, 2010). As in the analyses of eye movements, sensitivity to agent length was poor in sentences with the most complex patient descriptions.

In sum, these results show modulation of speech onsets by a number of variables: the presence of one or two patients (and thus the need for modification), and the ease of encoding the agent name and producing the agent NP. Thus, by the time that speakers initiated articulation, their planning of what they were going to say extended well beyond the first‐mentioned character.

### 5.3 Implications

The results have at least two implications for theories of message planning in language production. First, sensitivity to the complexity of the sentence‐final character suggests that message‐level planning scope can be very broad, consistent with Hierarchical Incrementality. In principle, speakers may have chosen a more efficient way of planning messages and their corresponding sentences by using a linearly incremental strategy of planning information specific to each character shortly before producing its name: that is, they could have prioritized encoding of agents before speech onset and then encoded information specific to the patient in a separate increment after speech onset. The fact that speakers showed early sensitivity to the manipulation of patient number and complexity (in the 200–600 ms time window) provides evidence against such a radical “left‐to‐right” bias in message‐level planning. In other words, message‐level encoding priorities need not be strictly driven by sentence‐level encoding priorities. Interestingly, message‐level encoding priorities continued to influence planning during time windows traditionally associated with sentence‐level encoding (i.e., after 600 ms). This confirms that the differences in eye movements that were observed across conditions shortly after picture onset were not short‐lived attention‐capture effects driven merely by perceptual differences in the pictured events across conditions.

Second, including the patient character in an early message plan may involve encoding basic features that allow speakers to gauge the difficulty of naming it (*Hypothesis 2*) but not necessarily finer‐grained detail, such as information about properties that distinguish it from another character and that require encoding of a separate postnominal phrase (*Hypothesis 3*). This temporal separation between encoding basic identifying information and encoding complex modifiers may be a time‐saving and cost‐saving strategy: modifiers can be incorporated into a developing message on the fly (Brown‐Schmidt & Konopka, 2008, 2015), so during early message planning, it may be sufficient to encode a patient slot with a “tag” indicating that new conceptual information about the patient is to be added later in a separate increment. This is consistent with the priorities of a multi‐tiered system where, to distribute processing load, speakers encode higher‐level information (like character identities) before lower‐level information (like additional character‐specific information) that can be encoded in a separate increment (also see Christiansen & Chater, 2016; MacDonald, 2013).

Of course, the decision to delay encoding of modifier‐specific information in this experiment may have been driven by the fact that, by design, the modifiers expressed information that was not immediately relevant for encoding crucial relational information (e.g., information needed to select an appropriate verb and an appropriate syntactic structure). Under Hierarchical Incrementality, speakers encode a relational who‐did‐what‐to‐whom framework early in the planning process, where “did what” information is critical (Konopka, 2019; Kuchinsky & Bock, 2010). If so, then any modifiers needed to provide additional detail on the “to whom” element in this framework can be encoded in a separate increment without detriment to the potential completeness of the early message plan. It is possible that, if the additional detail conveyed by modifiers were central to encoding “did what” information or if expressing it required encoding of grammatical dependencies, speakers may have devoted more early attentional resources to modifier‐specific information. This remains a question for future research. On the other hand, what constitutes crucial information in a message can change from context to context. Here, the presence of two patient characters in two‐patient events may have globally shifted the focus from early priority encoding of “did what” information to encoding of “to whom” information. The fact that encoding of detailed modifier information was nevertheless postponed suggests that it was more efficient for speakers to plan this information in a separate increment later in the production.

These results also raise an interesting question about the potential for flexibility and the boundaries of such flexibility in message planning. Previous research showed evidence of strategic choices during production of longer utterances, suggesting that speakers can choose to encode larger or smaller increments in order to flexibly distribute processing load over time (e.g., Ferreira & Swets, 2002). In the current study, the trade‐offs observed between planning agents and patients named in shorter or longer NPs showed that sensitivity to agent NP length was observed primarily in sentences with patients up to seven syllables in length. This shows that early message planning can be quite broad and that speakers can strategically choose to allocate more resources to one character versus another depending on encoding difficulty. Importantly, however, sentences with longer patient NPs showed no further modulation by agent NP length. In other words, generating a broad message plan is costly and there may be a limit to how much information can be encoded in parallel: speakers may, in principle, choose to allocate more early attention to one element of the message (e.g., the patient) but this reduces the likelihood of encoding information about other elements of the message (e.g., the agent) to the same extent. For present purposes, it is crucial that these decisions need not be driven by the “left‐to‐right” order of planning sentence‐level information, but they do suggest that there may be a limit to the upper bound of message‐level planning increments during spontaneous production.^6^

This upper bound is most likely driven by a combination of production‐specific time constraints (such as the requirement to produce agents first in active SVO sentences in English) and domain‐general cognitive constraints on the amount of information that can be planned in parallel in a given time window. Additionally, speaking often involves a trade‐off between two competing goals: the goal to plan ahead in order to maintain fluency and the goal to begin speaking quickly, with the latter reducing the amount of information that can be planned in advance. Arguably, incrementality—as a basic property of the production system—provides an efficient solution to this problem (Christiansen & Chater, 2016; MacDonald, 2013). If speakers are able to begin articulation before having planned everything they want to say, and if there is some degree of interleaving of “thinking” and “speaking,” then this reduces the pressure to prepare extensive messages in the first place. As such, support for *Hypothesis 3* may be difficult to find.

Finally, as outlined in the Introduction, another approach to answering questions about the breadth of planning comes from cross‐linguistic comparisons of the time‐course of planning. This research has examined the extent of early planning of message elements that are produced in sentence‐initial, sentence‐medial, and sentence‐final positions in languages that allow some word order variation (unlike English). In these languages, tracking the planning of characters that are produced in sentence‐final positions may be as problematic as it is for a language like English, as sentence‐level pressures (i.e., a “left‐to‐right” bias at the sentence level) can mask the effects of early planning of the sentence‐final character. The exception are cases of languages that explicitly mark grammatical dependencies between event characters, which drives more early fixations to both event characters regardless of their linear sentence placement (e.g., Sauppe et al., 2013). It remains to be established how much detail about individual characters is encoded during these early grammar‐driven gazes, i. e., whether speakers encode event role information alone and leave encoding of any more detailed character‐specific information to a later increment (with its timing being defined by word order) or not. Another solution is to test how verb placement influences early planning (e.g., Egurtzegi et al., 2022; Hwang & Kaiser, 2015; Momma, Slevc, & Phillips, 2016; Momma & Yoshida, 2023; Nordlinger et al., 2022; Sauppe, 2017). However, while there is evidence to suggest that encoding of sentence‐level verb information can be driven by word order, it is not clear again what type of verb‐specific information is planned early on. For example, it is possible that the early message‐level plan includes information about one character pushing another, but such an action can be expressed with a range of verbs, like *push* or *shove*, which express semantic nuances that may or may not be part of the original message plan (i.e., nuances that the current study aimed to track for a sentence‐final character with a modifier manipulation). Finally, any interpretations of the link between linear word order, grammatical requirements, and eye movement patterns must also take into account the degree of syntactic flexibility afforded by the target language. Variations in early eye movements, and thus differences in the extent to which a given message element is planned early on, cannot be completely dissociated from the process of selecting a syntactic structure or a specific linear word order, as these decisions can be influenced by a combination of variables (but see Sauppe & Flecken, 2021). This complicates the task of finding linguistic “minimal pairs” that allow for targeted comparisons of the planning of specific message elements while holding everything else constant.

## 6 Conclusions

In summary, planning scope at the message level in English can be extensive, encompassing high‐level (but not necessarily low‐level) information about message elements that are produced in early *as well as* late sentence positions. This finding is consistent with theories of incremental planning that assume the generation of a broad message‐level framework rather than the generation of a sequence of small message‐level increments. While some message elements in this broad framework may be more likely candidates for earlier sentence positions (e.g., the subject slot) than others, encoding priorities at the message level need not be immediately constrained by a likely linear word order.

## Conflict of interest statement

I have no conflicts of interest to disclose.

## Ethics statement

Ethical approval was obtained from the School of Psychology Ethics Committee.

## Supporting information



Appendix A
Supplementary Materials 1. Results of logistic mixed‐effects analyses of patient‐directed fixations in the 200‐600 ms, 600‐1800 ms, and 1800‐3000 ms time windows.

Supplementary Materials 2

## Data Availability

Data, scripts, and sample stimuli are available at: https://osf.io/rtjd3/.

## Notes

1 The terms “word‐by‐word” and “left‐to‐right” are used interchangeably to refer to a planning strategy where concepts and words are planned in an order that matches the linear word order of spoken utterances.

2 A key premise of both theories is that speakers try to output the material they have planned as soon as this material becomes available at the sentence level to reduce the need to buffer information in working memory. Accordingly, in lab experiments, speakers are asked to begin speaking as soon as they can, and differences in speech onsets across utterances and conditions are interpreted in terms of the amount of information that speakers were able to plan up to that point. In everyday language production, it is of course possible for speakers to plan what they want to say in full and to *not* start production immediately. Speakers may, for example, delay the onset of speech for a number of reasons or they may abandon or revise the original plan before beginning articulation. Current theories do not make predictions about the degree to which speakers use such a plan‐and‐revise strategy before initiating articulation under conditions of no time pressure (but see Gerwien, Schlenter, Penke, & Konopka, 2024, for evidence from preview paradigms).

3 Relational information can be expressed linguistically via lexical choices as well as structural choices. For present purposes, the examples focus on information expressed with verbs, as only one structural option was considered for the target sentences in the current study.

4 For present purposes, this category consisted of two types of agent‐first sentences: sentences with transitive verbs (e.g., *The child is picking up the bear*) and sentences with intransitive verbs (e.g., *The child is playing with the bear*).

5 The inclusion of multiple variables, and testing for high‐level interactions between these variables, outstrips the possibilities currently afforded by methods such as GAMM (see Ito & Knoeferle, 2023, for a review of different approaches to analyzing eye movement data). Assessing nonlinearities in agent‐directed fixations in the selected time window is not key to testing Hypotheses 1−3, as the predictions of these hypotheses differ with respect to the overall likelihood of speakers fixating agents and patients.

6 A plausible option for planning complex messages is, in fact, to assume that, within a single, “macro” message‐level increment that includes rudimentary information about all conceptual elements of the message (who‐did‐what‐to‐whom), there may be temporal differences in the order in which these individual “micro” elements (“who,” “did‐what,” “to‐whom”) are encoded. Hierarchical Incrementality proposes that encoding a complex hierarchical representation occurs via priority encoding of *relational* information, but this proposal does, in principle, leave open the possibility that non‐relational information may be encoded in separate smaller increments: “who” may receive priority over “to‐whom” and “to‐whom” may receive priority over “who” under different conditions. Testing this hypothesis requires tracking the timing of planning such “micro” increments with high precision.
