# Supplementary material for: Scope of Message Planning: Evidence From Production of Sentences With Heavy Sentence‐Final NPs
Source: Cogn Sci. 2025 Oct 14;49(10):e70110. doi: 10.1111/cogs.70110 (PMC12519050; doi:10.1111/cogs.70110)
Supplement: Supplementary file 1 — Appendix A Supplementary Materials 1. Results of logistic mixed‐effects analyses of patient‐directed fixations in the 200‐600 ms, 600‐1800 ms, and 1800‐3000 ms time windows. [file COGS-49-e70110-s001.docx]

Appendix A.

List of target items in alphabetical order. The expected pre-nominal and post-nominal modifiers required to describe the target patient in two-patient events are provided in parentheses.

| The baby is biting a (brown) bear (with green boots). |
| --- |
| The bee is stinging a (young) man (holding a cigar). |
| The bird is puncturing a (red) balloon (with a man hanging from it). |
| The boxer is punching a (short) man (holding a trophy). |
| The boy is kicking a (muddy) ball (with clovers). |
| The bride is cutting a (big) cake (with a figurine on top). |
| The burglar is opening a (brown) window (with a plant). |
| The cat is catching a (brown) mouse (next to the cheese). |
| The cat is scratching a (red) sofa (with a pillow). |
| The child is building a (blue/green) house (with a tower). |
| The children are dragging a (live) Christmas tree (with pinecones). |
| The cowboy is catching a (light) bull (with a nose ring). |
| The diver is pushing a (Asian) man (with a camera). |
| The doctor is feeding a (naked) baby (lying in a basket). |
| The dog is catching a (blue) butterfly (above a flower). |
| The dog is chasing a (blue) postman (riding a bike). |
| The dog is licking a (naked) baby (holding a bottle). |
| The elephant is lifting a (green) clown (holding a balloon). |
| The farmer is washing a (red) tractor (with a trolley). |
| The farmer is pulling a (large) donkey (with a basket). |
| The fireman is saving a (falling) boy (wearing a helmet). |
| The gardener is watering a (green) cactus (with a flower). |
| The girl is beating a (brown) rug (with a sunflower pattern). |
| The girl is opening a (yellow) gift (with hearts). |
| The girl is hanging a (pink) t-shirt (with ladybugs). |
| The girl is hugging a (brown) bear (holding a heart). |
| The girl is pulling a (brown) suitcase (with a cat on top). |
| The girl is taking a (brown) cake (with a candle). |
| The girl is trying on (black) boots (with laces). |
| The man is shooting a (red) apple (on top of a hat). |
| The horse is kicking a (thin) man (holding a rake).  The Inuit is attacking a (white) bear (holding a fish). |
| The judge is accusing a (blond) woman (holding a baby). |
| The magician is hypnotizing a (purple) clown (sitting in a wheelbarrow). |
| The man is brushing a (light) dog (who won 1st place). |
| The man is brushing a (grey) suit (with a pineapple pattern). |
| The man is chopping a (long) log (with a squirrel sitting on top). |
| The man is fixing a (old) tv (with an antenna). |
| The man is lifting a (long) bench (with a bucket). |
| The man is painting a (single) door (with hooks). |
| The man is planting a (upright) tree (with leaves). |
| The man is pushing a (purple) car (with a surfboard on top). |
| The man is threatening a (old) woman (with a shopping trolley). |
| The man is shooting a (young) woman (with an umbrella). |
| The monkey is taking the (brown) bananas (on a blanket). |
| The owl is taking a (brown) suitcase (with a tag). |
| The photographer is filming a (blond) model (wearing a hat). |
| The pirate is digging up a (brown) treasure chest (with a snake next to it). |
| The policeman is arresting a (blond) man (with a suitcase). |
| The punk is kicking a (tall) fence (with graffiti). |
| The robot is crushing a (grey) computer (on a table). |
| The sculptor is making a (brown) statue (with a bird on top). |
| The shark is attacking a (small/bald) man (with a life ring). |
| Superman is holding onto a (pink) motorbike (with a windshield). |
| The tailor is cutting a (long) dress (with sleeves). |
| The thief is stealing a (big) painting (of a windmill). |
| The tiger is scratching a (old) man (with a backpack). |
| The waiter is kicking out a (bald) chef (holding a pan). |
| The woman is breaking a (brown) vase (with flowers). |
| The woman is chasing a (large) chicken (carrying a hat). |
| The woman is cleaning a (wide) staircase (with a girl sitting at the top). |
| The woman is tickling a (smaller) girl (sitting on a pillow). |
|  |

Supplementary Materials 1. Results of logistic mixed-effects analyses of patient-directed fixations in the 200-600 ms, 600-1800 ms, and 1800-3000 ms time windows.

|  |  |  |  |  |  |  |  |  |  |  |  |  |  |  |  |  |  |  |
| --- | --- | --- | --- | --- | --- | --- | --- | --- | --- | --- | --- | --- | --- | --- | --- | --- | --- | --- |
|  |  | *b* | *SE* | *t* | *p* | *χ2* |  | *b* | *SE* | *t* | *p* | *χ2* |  | *b* | *SE* | *t* | *p* | *χ2* |
|  |  |  |  |  |  |  |  |  |  |  |  |  |  |  |  |  |  |  |
|  |  |  |  |  |  |  |  |  |  |  |  |  |  |  |  |  |  |  |
| 200-600 ms time window | | | | | | |  | 600-1800 ms time window | | | | |  | 1800-3000 ms time window | | | | |
| Fixed effects |  |  |  |  |  |  |  |  |  |  |  |  |  |  |  |  |  |  |
| Intercept |  | -.43 | .16 | -2.62 | <.01 | -- |  | -.86 | .08 | -10.53 | <.0001 | -- |  | .09 | .08 | 1.08 | .28 | -- |
| Patient Number |  | .42 | .19 | 2.22 | .03 | 1.95 (.16) |  | .14 | .12 | 1.10 | .23 | **.03 (.87)** |  | **-.07** | **.16** | **-.44** | **.66** | **2.91 (.09)** |
| Patient Complexity |  | **.79** | **.16** | **5.01** | **<.0001** | **17.49 (<.0001)** |  | **.34** | **.09** | **3.73** | **<.001** | **18.01 (<.0001)** |  | **.25** | **.12** | **2.09** | **.04** | **6.23 (.01)** |
| Patient Codability |  | -.12 | .16 | -.75 | .45 | .83 (.36) |  | .04 | .07 | .57 | .57 | .37 (.54) |  | .03 | .07 | .46 | .65 | .22 (.64) |
| Patient Length (syllables) |  | **-.20** | **.08** | **-2.38** | **.02** | **7.33 (<.01)** |  | -.03 | .06 | -.47 | .64 | .03 (.86) |  | .11 | .08 | 1.34 | .18 | .38 (.54) |
| Patient Number * Patient Complexity |  | .20 | .24 | .84 | .40 | 3.37 (.07) |  | .15 | .19 | .82 | .41 | .83 (.36) |  | .17 | .25 | .70 | .48 | 1.29 (.26) |
| Patient Number * Patient Codability |  | -.05 | .11 | -.52 | .60 | .48 (.49) |  | .01 | .07 | .21 | .83 | .04 (.84) |  | -.11 | .10 | -1.18 | .24 | 2.23 (.14) |
| Patient Number * Patient Length |  | .14 | .07 | 1.91 | .06 | 2.19 (.14) |  | **.26** | **.04** | **6.43** | **<.0001** | **41.17 (<.0001)** |  | **.16** | **.04** | **4.19** | **<.0001** | **15.73 (<.0001)** |
| Patient Complexity * Patient Codability |  | -.15 | .11 | -1.33 | .18 | 1.50 (.22) |  | -.04 | .06 | -.70 | .49 | .50 (.48) |  | -.10 | .07 | -1.44 | .15 | 3.30 (.07) |
| Patient Complexity * Patient Length |  | -.08 | .07 | -1.05 | .29 | 12.47 (<.001) |  | **-.17** | **.04** | **-4.41** | **<.0001** | **29.50 (<.0001)** |  | **-.47** | **.04** | **-12.29** | **<.0001** | **274.69 (<.0001)** |
| P. Number * P. Complexity * P. Codability |  | **-.34** | **.17** | **-2.07** | **.04** | **4.27 (.04)** |  | .00 | .11 | .03 | .98 | .00 (.98) |  | .31 | .16 | 1.93 | .053 | 3.73 (.053) |
| P. Number * P. Complexity * P. Length |  | **-.55** | **.14** | **-3.87** | **<.001** | **14.98 (<.001)** |  | -.04 | .08 | -.48 | .63 | .23 (.63) |  | **-.29** | **.08** | **-3.79** | **<.001** | **14.33 (<.001)** |
| Random effects |  | Var. | SD | Corr. |  |  |  | Var. | SD | Corr. |  |  |  | Var. | SD | Corr. |  |  |
| Items (intercept) |  | 1.43 | 1.20 |  |  |  |  | .29 | .54 |  |  |  |  | .24 | .49 |  |  |  |
| Patient Number |  | .59 | .77 | -.12 |  |  |  | .23 | .48 | -.21 |  |  |  | .51 | .72 | -.10 |  |  |
| Patient Complexity |  | .70 | .83 | .07 .32 | |  |  | .19 | .43 | .00 -.11 | |  |  | .28 | .53 | -.09 .12 | |  |
| P. Number * P. Complexity |  | 1.23 | 1.11 | -.13 -.16 .01 | |  |  | .56 | .75 | .03 .05 .17 | |  |  | 1.35 | 1.16 | -.12 .13 .17 | |  |
| Participants (intercept) |  | .15 | .39 |  |  |  |  | .09 | .31 |  |  |  |  | .12 | .35 |  |  |  |
| Patient Number |  | 1.13 | 1.06 | .30 |  |  |  | .45 | .67 | .03 |  |  |  | .86 | .93 | .31 |  |  |
| Patient Complexity |  | .53 | .73 | .20 .69 | |  |  | .22 | .47 | .09 .54 | |  |  | .42 | .65 | .25 .68 | |  |
| Patient Length |  | .29 | .54 | -.11 -.84 -.73 | |  |  | .18 | .43 | .00 -.89 -.49 | |  |  | .33 | .57 | -.22 -.84 -.67 | |  |
| Patient Codability |  | .07 | .25 | .16 -.11 -.11 .14 | | |  | .03 | .17 | .03 .21 .46 -.27 | | |  | .06 | .25 | -.35 -.32 -.20 .33 | | |
| P. Number * P. Complexity |  | 1.14 | 1.07 | .09 .45 .49 -.43 -.14 | | |  | 1.14 | 1.07 | -.14 .53 .34 -.62 .14 | | |  | 1.77 | 1.33 | .28 .71 .42 -.77 - .27 | | |
|  |  |  |  |  |  |  |  |  |  |  |  |  |  |  |  |  |  |  |

Supplementary Materials 2. Model fits for the three-way interactions with Agent Codability in each time window, shown separately for simple-patient and complex-patient events.

ADD FIGURE SUPPLEMENTARY MATERIALS 2 HERE
