# Supplementary material for: Scope of Message Planning: Evidence From Production of Sentences With Heavy Sentence‐Final NPs
Source: Cogn Sci. 2025 Oct 14;49(10):e70110. doi: 10.1111/cogs.70110 (PMC12519050; doi:10.1111/cogs.70110)
Supplement: Supplementary file 2 — Supplementary Materials 2 [file COGS-49-e70110-s002.pdf]

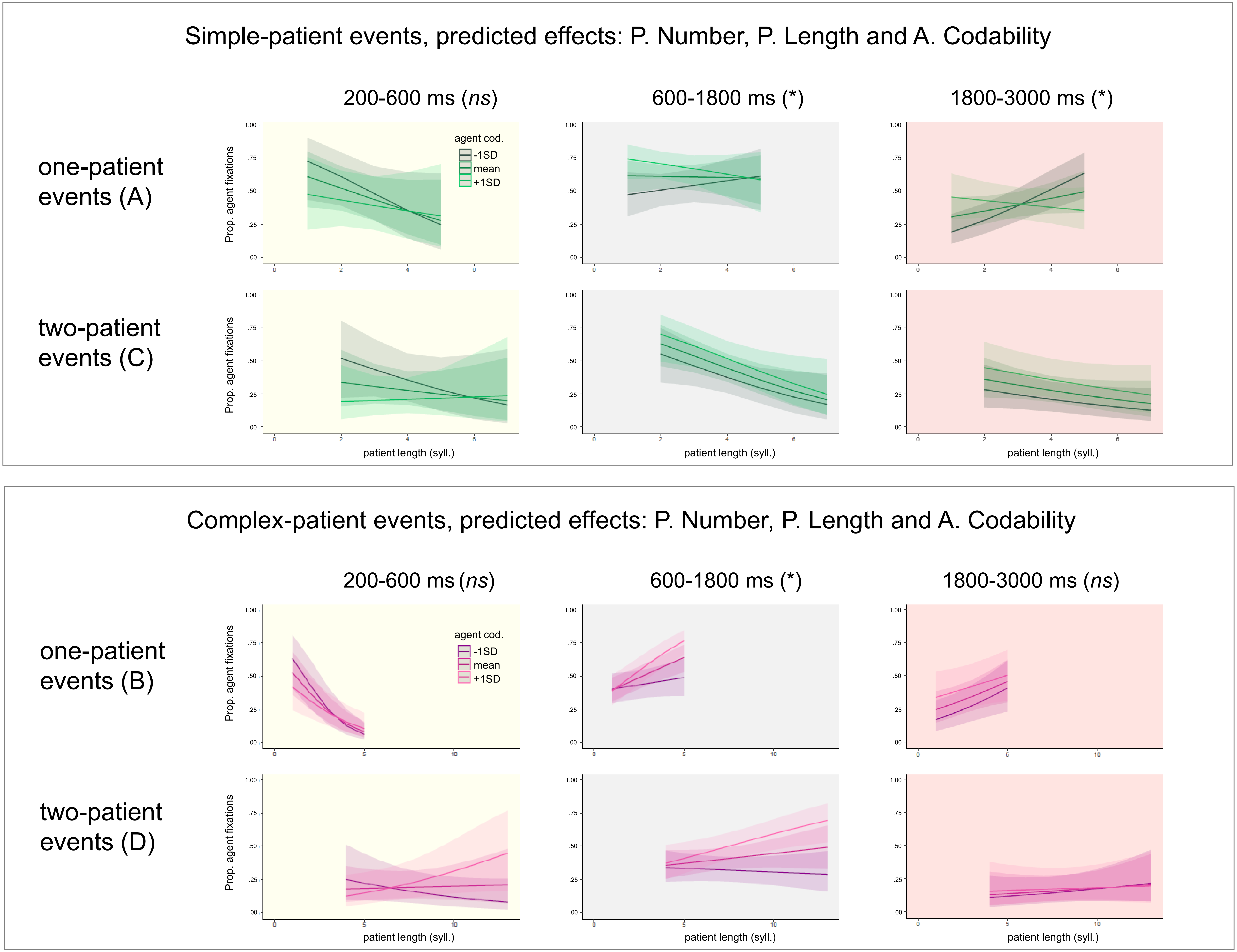

Simple-patient events, predicted effects: P. Number, P. Length and A. Codability
200-600 ms (ns)
600-1800 ms (*)
1800-3000 ms (*)
one-patient events (A)
two-patient events (C)
Prop. agent fixations
agent cod.
-1SD
mean
+1SD
patient length (syll.)
Complex-patient events, predicted effects: P. Number, P. Length and A. Codability
200-600 ms (ns)
600-1800 ms (*)
1800-3000 ms (ns)
one-patient events (B)
two-patient events (D)
Prop. agent fixations
agent cod.
-1SD
mean
+1SD
patient length (syll.)
